# The role of self-management in endometriosis pain: insights from a cross-sectional survey in Germany, Austria, and Switzerland

**DOI:** 10.1007/s00404-025-08019-1

**Published:** 2025-04-19

**Authors:** Franziska Werner, Victoria Jasinski, Renata Voltolini Velho, Jalid Sehouli, Sylvia Mechsner

**Affiliations:** Department of Gynaecology Charité With Centre of Oncological Surgery, Endometriosis Research Centre Charité, Campus Virchow-Klinikum, Augustenburger Platz 1, 13353 Berlin, Germany

**Keywords:** Endometriosis, Self-management, Pain management, Non-pharmacological treatment

## Abstract

**Background:**

Endometriosis has a significant negative impact on women’s lives. Unfortunately, current medical treatments often fail to provide adequate pain relief and may cause intolerable side effects. Although many women experiencing primary dysmenorrhoea employ self-management strategies to help alleviate period-related symptoms, there is a paucity of knowledge about how women with endometriosis manage their symptoms through self-management.

**Methods:**

A cross-sectional online survey was distributed in Germany, Austria, and Switzerland, between August and December 2022, targeting women aged 18 years or older with a diagnosis of endometriosis. The survey gathered information on (pharmacological and non-pharmacological) self-management strategies employed by the respondents in the previous six months, including their frequency, reasons for non-use, self-rated effectiveness, and impact on reducing endometriosis-related medication. Furthermore, the survey collected data on demographics, medical history, current symptomatology, and medication usage. Descriptive statistical analyses were conducted.

**Results:**

Of the 912 valid responses, 75.4% reported using self-management strategies, with the most prevalent being rest (91.6%), heat (91.1%), and exercise (63.3%). The most highly rated techniques in terms of effectiveness in pain reduction were cannabis, osteopathy, heat, and alcohol, with mean effectiveness ratings of 8.0, 7.3, 7.1, and 6.8, respectively, on the Numerical Rating Scale. Interventions, such as Tai Chi/Qi Gong, yoga/Pilates, herbal medicine, stretching, and meditation/breathing, were rated as being less effective. The lack of information and costs were identified as the primary reasons for not utilising self-management approaches.

**Conclusion:**

The findings of this study may provide evidence for the reimbursement of self-management techniques by health insurance companies for the treatment of endometriosis-associated pain.

**Supplementary Information:**

The online version contains supplementary material available at 10.1007/s00404-025-08019-1.

## What does this study add to the clinical work

**Table:** 

This study provides comprehensive overview of self-administered and therapist-assisted self-management strategies used by individuals with endometriosis, highlighting their perceived effectiveness. The findings support clinicians in providing evidence-informed, individualised recommendations for non-pharmacological pain management based on patients' preferences and resources.	

## Introduction

It is estimated that 39.6% of women are affected by gynaecological diseases with pain as a central symptom [1]. These include endometriosis, a benign chronic inflammatory disease in which endometrial-like tissue implants and grows outside the uterine cavity, particularly in the pelvic peritoneum, ovaries, and rectovaginal septum. This condition presents a spectrum of symptoms, with dysmenorrhoea, dyspareunia, dysuria, dyschezia, and cyclical and acyclical pelvic pain being the cardinal complaints, along with bleeding disorders as well as infertility. Beyond the immediate symptoms, the burden of endometriosis encompasses the repercussions of these symptoms on various facets of women's lives. The impact of endometriosis symptoms spans daily activities, appearance, emotional well-being, financial impacts, physical activity, sex-related impacts, sleep, social engagements, and work or school commitments. Affecting 6–10% of women of reproductive age [2, 3], endometriosis is as prevalent as other chronic diseases, such as diabetes mellitus [4]. However, the average time between the onset of symptoms and diagnosis is 10.4 years, due to misdiagnosis, the normalisation of pelvic pain by healthcare professionals, and the use of hormonal contraception to suppress symptoms. The duration of diagnosis has been identified as a contributing factor to the development of endometriosis-related symptoms, comorbidities, and pain chronification [5].

In the absence of a causal therapy to date, the current first-line treatment consists of hormonal and surgical treatment, with pain being treated with non-steroidal anti-inflammatory drugs (NSAIDs) and, in severe cases, opioids. It has been demonstrated that more than 30% of patients are unable to achieve pain relief from conventional medical and surgical therapies for endometriosis [6]. Despite improving endometriosis symptoms, the challenges of hormonal contraceptives include side effects such as reduced oestrogen levels potentially leading to, and mood swings [7]. Additionally, they are not suitable for those who are trying to conceive. Furthermore, NSAIDs are associated with an increased risk of gastrointestinal bleeding, whilst metamizole has been linked to hepatotoxicity. The prescription of opioids should be closely supervised due to the risk of addiction [8, 9].

Self-management strategies, including dietary products, acupuncture, yoga/Pilates, and electrotherapy are not yet considered as therapeutic options, and their costs are not covered by health insurance due to insufficient evidence [4, 10, 11]. Although most women experiencing primary dysmenorrhoea rely on self-care techniques and lifestyle choices to alleviate menstrual symptoms [12, 13], there is limited information available about how women with endometriosis cope with their symptoms through self-management [2].

These data have the potential to support the inclusion of self-management techniques for endometriosis pain in health insurance coverage.

## Methods

### Study design

The design of the online questionnaire was based on a previous Australian national study on self-management strategies amongst individuals affected by endometriosis, conducted by Armour et al. [14]. The survey was carried out online between August 1^st^ and December 31^st^, 2022 in Germany, Austria, and Switzerland, with participants requiring between 15 and 40 min to complete the questionnaire. A full copy of the survey is available in the Supplementary Material 1.

In this study, self-management strategies were defined as all non-pharmacological interventions used by individuals with endometriosis to alleviate symptoms. However, these strategies can be categorised into two distinct groups: (1) self-directed techniques that individuals can apply independently, including heat, cold, stretching, exercise, dietary changes, and relaxation techniques (e.g., yoga/Pilates), and (2) externally administered interventions requiring a therapist, such as osteopathy, acupuncture, and massage. Whilst both categories contribute to the management of symptoms, it is important to note that the latter often involves additional costs and accessibility barriers, which may influence their usage. Furthermore, herbal medicine, including cannabis products, can be regarded as a discrete category of alternative medicine rather than a behavioural self-management approach. This distinction provides a more structured perspective on the range of interventions available and their potential impact on accessibility and cost.

The objective of the questionnaire was to gather information on the self-management strategies employed by respondents in the past six months, whether pharmacological or non-pharmacological. This included an investigation of the frequency of use and reasons for non-use, self-rated effectiveness, and the potential impact on reducing endometriosis-related medication. Furthermore, the survey gathered data on the respondent’s demographic characteristics, diagnostic history, current symptomatology, and medication usage.

The data generated from the question regarding current hormone treatment were initially analysed using Microsoft Excel v16.71, and subsequently subjected to descriptive statistical analyses. To evaluate endometriosis-associated pain and the self-rated effectiveness of self-management methods, an 11-point numerical rating scale (NRS) from 0 to 10 was employed. In this context, a rating of 0 indicates the absence of pain or ineffectiveness, whereas a rating of 10 indicates the strongest pain or highest level of effectiveness. The evaluation of the impact of pelvic pain on different aspects of life was conducted using a 5-point scale, where a rating of 1 indicates no impact and a rating of 5 indicates a significant impact.

### Study population and recruitment

The participants for the study were recruited though a variety of sources, including the Endometriosis Centre Charité, the Endometriosis Association in Germany, Austria, and Switzerland, as well as social media platforms. Participants were eligible in the survey if they were 18 years or older, premenopausal, German-speakers, currently living in Germany, Austria, and/or Switzerland, and had a diagnosis of endometriosis. Individuals suspected of having endometriosis or those under 18 years of age were excluded from participation.

### Sample size

To ensure that the results are statistically significant for the population of individuals affected by endometriosis in German-speaking countries, a sample size calculation was carried out based on the research by Serdar et al. [15]. There are an estimated 3,108,000–5,180,000 women affected by endometriosis in Germany, Austria, and Switzerland, which represents 6–10% of the total female population in these countries [16–18]. This estimation resulted in a required sample size of 385 participants. The calculation considered a margin error of 5%, a confidence level of 95%, and a resulting *z*-score of 1.96. Since the questionnaire has multiple questions, the value of p was set at 0.5 to determine the largest required sample size for the selected confidence level and margin of error.

### Statistical analysis

The statistical analyses were conducted using IBM SPSS Statistics (Version 29.0.0.0). Missing data were not replaced during the analyses. Descriptive statistics were employed, presenting continuous data as means with standard deviations (SD) and categorical data as relative frequencies with percentages. Correlation analyses were conducted using Spearman’s correlation with statistical significance set at *p* < 0.05. The effect size was determined based on the correlation coefficient (*r*), with effect cut-offs defined according to Cohen as weak (0.1–0.3), moderate (0.3–0.5), and strong (*r* > 0.5) [19].

## Results

### Demographic characterisation

A total of 915 women completed the survey, resulting in a response rate of 65.2%. Three responses were excluded from the analyses, since the participants had not consented to participate, were outside the age range specified, or had not been diagnosed with endometriosis. A total of 912 responses were considered suitable for inclusion in the subsequent analysis.

For the question about hormone treatment, non-specific answers were excluded from the analysis.

The mean age of the responding women was 30.6 years (± 6.5), with 94.9% residing in Germany (Table 1). The majority of participants (82.7%) were employed and in a stable relationship (48.9%).

**Table 1:** Demographic characteristics of participants

	Mean/number	SD/percentage	*N*
Age (years)	30.6	6.6 SD	891
18–24	158	17.3	
25–29	273	29.9	
30–34	241	26.4	
35–39	127	13.9	
40–44	68	7.5	
45–49	18	2.0	
50 +	6	0.7	
Country			909
Germany	863	94.9	
Austria	31	3.4	
Switzerland	15	1.6	
Employment			911
Employed	754	82.7	
Unemployed	45	4.9	
Housewife	21	2.3	
Retired	18	2.0	
None of the above	73	8.0	
Net Income			912
No own income	50	5.5	
under 500€	52	5.7	
500€ to under 1000€	102	11.2	
1000€ to under 1500€	143	15.7	
1500€ to under 2000€	163	17.9	
2000€ to under 2500€	234	25.7	
2500€ to under 3000€	91	10.0	
≥3000€	77	8.4	
Current marital status			912
Single	176	19.3	
Single parent	22	2.4	
Married/registered civil partnership	264	28.9	
In a steady relationship	446	48.9	
Divorced	16	1.8	
Other	7	0.8	

### Clinical characterisation

The mean delay for diagnosing endometriosis was 9.2 years (± 6.6), with 88.9% receiving a diagnosis through laparoscopy or other surgery (Table 2). On average, participants underwent 1.8 (± 1.3) surgeries, and 61.3% of the surgeries were performed in endometriosis-certified centres. Furthermore, the mean Revised American Society for Reproductive Medicine (rASRM) stage was 2.5 (± 1.1), and 117 participants also suffered from adenomyosis.

**Table 2:** Clinical characterisation of participants’ history regarding endometriosis

	Mean/number	SD/percentage	*N*
Diagnostic delay (years)	9.2	6.6 SD	875
Diagnosis			912
Laparoscopy/surgery	811	88.9	
MRT	13	1.4	
Sonography	59	6.5	
Other	29	3.2	
Surgeries	1.8	1.3 SD	772
1	458	59.3	
2	179	23.2	
3	56	7.3	
4	29	3.8	
5	20	2.6	
> 5	30	3.9	
rASRM stage	2.5	1.1 SD	768
Not specified	310	5.5	
I	127	5.7	
II	143	11.2	
III	124	15.7	
IV	113	17.9	
ENZIAN classification			751
Not specified	527	70.2	
A1	31	4.1	
B1	47	6.3	
C1	22	2.9	
A2	33	4.4	
B2	67	8.9	
C2	18	2.4	
A3	29	3.9	
B3	30	4.0	
C3	20	2.7	
FA	117	15.6	
FB	25	3.3	
FU	13	1.7	
FI	27	3.6	
FO	24	3.2	
Number of hormone therapies			912
0	168	18.4	
1	222	24.3	
2	206	22.6	
3	128	14.0	
4	75	8.2	
5	22	2.4	
More than 5	91	10.0	
Current hormone therapy			729
None	263	36.1	
Combined oral contraceptive pill	112	15.4	
Progestogen-only pill	62	8.5	
Gestagen (dienogest/progesterone)	203	27.8	
GnRH analogues	1	0.1	
Hormone replacement therapy	5	0.7	
Intrauterine device	73	10.0	
Depo	11	1.5	
Vaginal oestrogen	5	0.7	
Other	10	1.4	
Bleeding free			908
Yes	368	40.5	
No	540	59.5	

Most participants (81.6%) received one or more hormonal medications for endometriosis treatment, with 64.7% currently undergoing hormone therapy, leading to 40.5% experiencing cessation of menstrual bleeding.

### Intensity of endometriosis-associated pain types and effect on daily life

The study findings showed that 99.5% of the participants experienced at least one of the listed symptoms (Table 3). Dysmenorrhoea was reported by 89.3%, cyclic pelvic pain by 86.7%, acyclic pelvic pain by 83.5%, dyspareunia by 75.0%, dyschezia by 71.8%, and dysuria by 49.5% of the respondents.

**Table 3:** Endometriosis-associated pain types, pain levels, and effect of pelvic pain on aspects of daily life

	Mean/number	SD/percentage	*N*
Endometriosis-associated symptoms			907
Cyclic pelvic pain	786	86.7	
Acyclic pelvic pain	757	83.5	
Dysmenorrhoea	810	89.3	
Dyspareunia	680	75.0	
Dyschezia	651	71.8	
Dysuria	450	49.6	
Pain level under hormones			
With bleeding	5.0	2.9	540
Without bleeding	4.2	2.5	368
Pain level under medication			
No hormone treatment	5.4	2.3	263
With bleeding	5.2	2.1	540
Without bleeding	5.3	2.3	368
Effect pelvic pain			899
Mood	3.9	1.0	
Exercise	3.7	1.2	
Sexual behaviour	3.6	1.3	
Digestion	3.6	1.1	
Productivity	3.5	1.0	
Daily life	3.5	1.1	
Wearing certain items of clothing	3.5	1.3	
Sleep	3.2	1.2	
Sitting	2.7	1.3	

Endometriosis-associated pain is presented as number of participants and percentage. Pain levels were rated by participants on the NRS (0–10) and the effect of pelvic pain on aspects of daily life on a 5-point scale. Values are shown as mean and SD

The study also presented the differences in pain levels between hormone treatment and analgesic medication, and within these categories, between patients with menstrual bleeding, and those where medication leads to a cessation of menstrual bleeding (therapeutical amenorrhoea, see Table 3). The lowest pain level was observed in patients undergoing hormone treatment with therapeutical amenorrhoea (4.2 ± 2.5), whilst the highest pain level was detected in patients not undergoing hormone treatment (5.4 ± 2.3). In the cohort undergoing hormone treatment with therapeutical amenorrhoea, 6.5% of participants reported a pain level of 0. Furthermore, 98.1% of the participants indicated that pelvic pain had an impact on at least one of the listed aspects of daily life. Over 50% of participants reported that their pelvic pain in the previous month had a significant impact on various aspects of their lives, including productivity, mood, digestion, daily activities, exercise, wearing certain items of clothing, and sexual behaviour. In particular, 97.4% of participants with dyspareunia experienced strain in their partnership due to endometriosis, and 95.3% stated that pelvic pain affected their sexual behaviour in the previous month.

Notably, a strong negative correlation was identified between the year of onset of endometriosis-associated symptoms and diagnostic delay (*r* = −0.839, *p* < 0.001, Table 4). Additionally, a weak positive correlation between diagnostic delay and the number of endometriosis-associated symptoms (*r* = 0.149, *p* < 0.001) was observed. The correlation between diagnostic delay and rASRM stage was not statistically significant. However, a weak correlation was identified between diagnostic delay and intensity of pain experienced under hormone therapy (*r* = 0.110, *p* = 0.001), whilst no correlation was detected between diagnostic delay and intensity of pain experienced under medication (*r* = 0.088, *p* = 0.009). Furthermore, no correlation between the rASRM stage and pain level could be identified.

**Table 4:** Correlation analyses

	*p*-value	*r*
Diagnostic delay and rASRM stage	0.051	0.072
Diagnostic delay and pain		
Under hormone treatment	0.001	0.110
Under analgesic medication	0.009	0.088
Diagnostic delay and number of endometriosis-associated symptoms	< 0.001	0.149
rASRM stage and pain		
Under hormone treatment	0.393	−0.040
Under analgesic medication	0.803	−0.012
Year onset symptoms and diagnostic delay	< 0.001	−0.839

### Use of analgesics

**Fig. 1:**
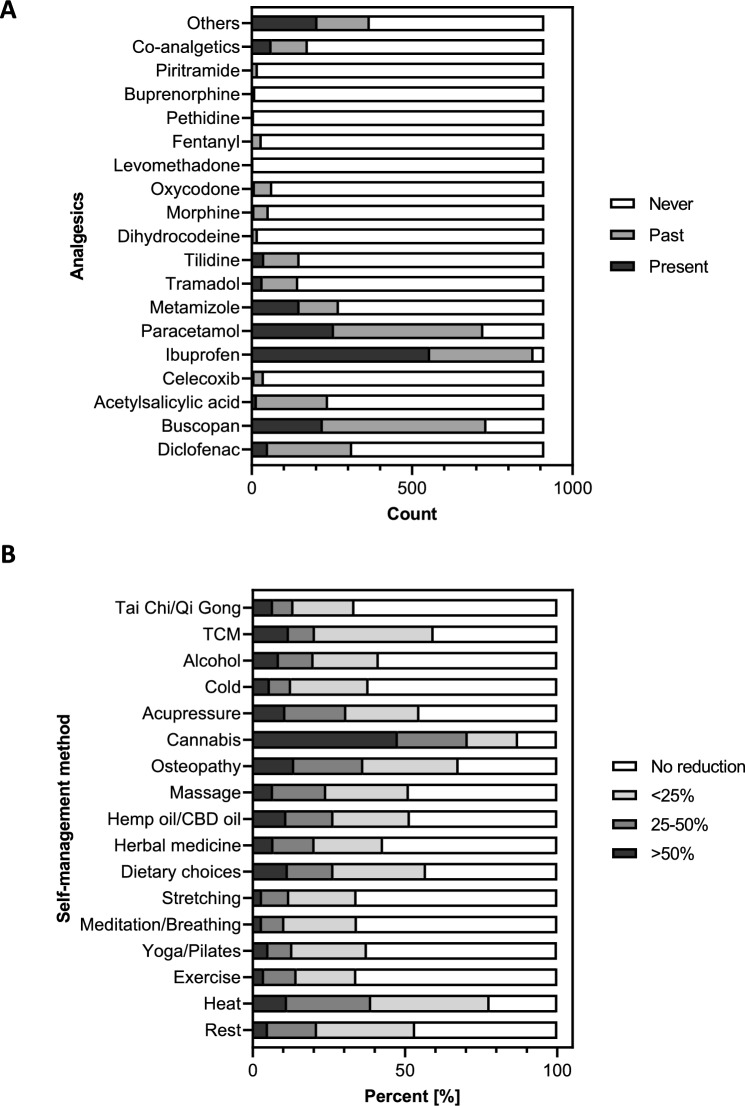
Analgesic usage and effect of self-management on medication reduction. **A** Use of analgesics against endometriosis-associated pain (*N* = 912). **B** Reduction of endometriosis-related medication under usage of self-management methods

A large majority of patients (96.4%) reported the use of ibuprofen, 80.3% used buscopan and 79.2% used paracetamol in the present or past to manage endometriosis-associated pain. The use of opioids, including tramadol (15.9%), tilidine (16.3%), and oxycodone (7%), is illustrated in Fig. 1A. 27.52% of the participants stated that they had used opioids in the past or were currently using them.

### Complementary and alternative therapies

A significant majority (75.4%) of endometriosis patients used self-management techniques within the last six months. The primary reasons for the non-utilisation of self-management strategies were a lack of information (47.9%), costs (39.7%), time commitment (37.9%), difficulty accessing resources (29.7%), previous ineffective experience (21.9%), and other factors (17.4%). The most frequently mentioned methods included rest (91.6%), heat (91.1%), exercise (63.3%), yoga/Pilates (54.2%), meditation or breathing exercises (54.1%), and stretching (53.9%) (Table 5).

**Table 5:** Use of self-management techniques amongst endometriosis patients, including number of patients who have used the respective method in the last six months (*N* = 687), effectiveness, and pain level after using the technique

Self-management technique	Use number (percentage)	Effectiveness Mean (SD)	Pain level Mean (SD)
Cannabis	114 (16.6)	8.0 (2.3)	4.2 (2.8)
Osteopathy	184 (26.8)	7.3 (2.7)	5.1 (2.3)
Heat	626 (91.9)	7.1 (2.0)	5.0 (2.2)
Alcohol	71 (10.3)	6.8 (3.3)	4.3 (2.4)
Dietary choices	270 (39.3)	6.4 (2.8)	5.0 (2.3)
Hemp oil/CBD oil	231 (33.6)	6.3 (3.0)	5.6 (2.1)
Rest	629 (91.6)	6.3 (2.5)	5.2 (2.1)
Massage	207 (30.2)	6.0 (2.5)	5.1 (2.1)
Cold	75 (10.9)	5.9 (2.7)	5.2 (2.4)
Acupressure	81 (11.8)	5.8 (3.1)	5.1 (2.5)
TCM	65 (9.5)	5.7 (3.0)	5.2 (2.4)
Exercise	435 (63.3)	5.6 (2.8)	4.6 (2.2)
Meditation/breathing	372 (54.1)	5.5 (2.7)	4.9 (2.2)
Stretching	370 (53.8)	5.5 (2.6)	4.9 (2.0)
Herbal medicine	233 (33.9)	5.3 (3.0)	5.4 (2.3)
Yoga/Pilates	386 (56.2)	5.1 (2.4)	4.2 (2.1)
Tai Chi/Qi Gong	15 (2.2)	4.9 (3.7)	4.3 (3.1)

Data were listed from greatest to smallest reported effectiveness

First, it is important to note that active self-management approaches, such as yoga/Pilates, Tai Chi/Qi Gong, and exercise, are unlikely to be performed in severe pain. Consequently, pain levels are presumably lower from the outset. Therefore, it is not possible to assume that a low pain level when performing such techniques necessarily indicates high effectiveness.

The lowest pain levels were observed in the use of cannabis (4.2 ± 2.8), followed by yoga/Pilates (4.2 ± 2.1) and Tai Chi/Qi Gong (4.3 ± 3.1). Conversely, the highest levels of pain were reported with the use of CBD/hemp oil (5.6 ± 2.1), herbal medicine (5.4 ± 2.3), and cold (5.2 ± 2.4, Table 5).

The most effective self-management strategies for reducing pelvic pain were identified as cannabis (8.0 ± 2.3), osteopathy (7.3 ± 2.7), and heat (7.1 ± 2.0) by the respondents. The effectiveness of Tai Chi/Qi Gong (4.9 ± 3.7), yoga/Pilates (5.1 ± 2.4), and herbal medicine (5.3 ± 3.0) was perceived to be less significant (Table 5). Amongst those who used cannabis, 47.7% reported a reduction in their endometriosis-related medication of over 50%, compared to 11.0% of those who used CBD/hemp oil. Furthermore, 22.9% of cannabis users reduced their medication by 25–50%, compared to 15.5% of CBD/hemp oil users. The remaining self-management strategies were found to be considerably less effective. Amongst women who used heat therapy, 11.3% reported a reduction in medication of over 50%, whilst osteopathy was the primary reason for a reduction in medication of over 50% for 13.6% of women (Fig. 1B).

The self-management methods with the lowest monthly costs in the study were cold therapy (0.3€ ± 1.2€), rest (3.2€ ± 21.2€), and stretching (5.6€ ± 23.0€). In contrast, acupressure (150.4€ ± 135.8€), Traditional Chinese Medicine (TCM; 126.3€ ± 113.4€), and osteopathy (115.8€ ± 91.9€) were the most expensive. With a monthly expense of 97.30€ (± 115.87€), cannabis is amongst the costlier self-management options.

## Discussion

This study presents, for the first time, a comprehensive overview of the utilisation of various pharmacological and non-pharmacological therapeutic options and the prevailing pain experiences amongst endometriosis patients in the German-speaking region of Central Europe.

The self-management method rated most effective in our study for alleviating endometriosis-associated pain was cannabis, which is consistent with the findings of other studies [14, 20]. It is noteworthy that 16.6% of those who employed self-management techniques reported using cannabis to manage their endometriosis-related pain over the previous six months. This is in spite of the fact that cannabis purchase, cultivation, and possession were illegal in Germany at the time of the survey. The prescription of medicinal cannabis is ruled by the National Association of Statutory Health Insurance (Kassenärztliche Bundesvereinigung-KV) and can be accessed by patients with serious diseases. Endometriosis is included as a potential indication for medicinal cannabis if other pain medications have already been exhausted and the patient is refractory to opioid treatment [21]. Furthermore, access to medical cannabis is constrained by societal and professional stigma. As in other studies, the heat was the only method in our survey that was both widely utilised and highly effective [14, 20]. It is notable that certain self-management techniques, such as osteopathy, massage, and TCM, lack substantial research, examining their effectiveness and mechanisms of action in the context of endometriosis. These methods serve to complement medical treatment, thereby facilitating a reduction in both pain and stress. Additionally, self-management techniques empower women to assume an active role in their treatment, thereby enhancing their quality of life.^2,9^ Our study demonstrated that the utilisation of self-management methods amongst endometriosis patients in German-speaking countries is markedly high at 75.4%. This is comparable to the findings of similar cross-sectional studies conducted in Australia (76%) [14] and Canada (93.8%) [20].

Successful self-management hinges on education and awareness of endometriosis. Numerous online resources, self-help groups, and organisations exist to support women in making informed decisions and effectively communicating with healthcare professionals. These resources provide invaluable information and guidance for living with the disease whilst also offering a safe space for patients to share their experiences, given that endometriosis remains stigmatised despite growing awareness. It is important to note that a significant number of self-management techniques are not covered by statutory health insurance due to a lack of evidence. Consequently, patients are frequently required to bear the associated costs themselves.

Pain management constitutes a pivotal element in the treatment of patients with endometriosis. Given the hormone-dependent nature of the disease, hormonal treatment represents the fundamental therapeutic approach, intending to achieve therapeutic amenorrhoea. Nevertheless, the present study revealed that 93.5% of the participants reported pain in the absence of menstrual bleeding under hormone treatment. In addition to hormonal treatment, the use of analgesics is recommended for acute nociceptive pain, as they suppress the release of pain neurotransmitters and possess anti-inflammatory properties. It is important to note that recent trends indicate an increasing reluctance amongst patients to utilise synthetic hormones, driven by concerns about their unnatural origin and potential harm to the body [4]. In our study, the combined treatment of hormonal therapy to induce amenorrhea in conjunction with additional medication yielded the lowest pain levels amongst medical treatments, particularly in comparison to pain medication alone without the use of hormones. However, with an average pain level of 5.3 ± 2.3, a considerable degree of discomfort persists under these circumstances, significantly limiting daily activities. These findings are corroborated by several studies [6, 22], including Zhao et al. [23], which highlighted the limited effectiveness and numerous side effects associated with conservative hormonal therapy.

Our study revealed a correlation between the year of onset of first symptoms and the diagnostic delay. Compared to a 2012 study conducted by Hudelist and colleagues [24], our results of 9.2 years on average confirmed the long and after 10 years almost unchanged diagnostic delay. However, this time frame is excessive and reflects a lack of awareness and normalisation of chronic pelvic pain both amongst healthcare professionals and in society [25]. Additionally, the aforementioned period is longer in Germany than in other countries, such as the United Kingdom (8 years) [26], The Netherlands (8.5 years) [25], or Canada (5.4 years) [27]. Our study identified a weak correlation between diagnostic delay and pain intensity, both in the context of hormone treatment and medication. Furthermore, a weak correlation was observed between diagnostic delay and the number of endometriosis-associated symptoms, which aligns with the results of a study conducted by Surrey and collaborators [5]. Early diagnosis and appropriate pain management, even before diagnosis, are critical to preventing the development of central sensitisation mechanisms that lead to the emergence of overlapping pain syndromes later [28]. Our study revealed that approximately two-thirds of participants underwent surgery at a certified endometriosis centre. However, subsequent treatment was typically delegated to their local gynaecologists, who are often not specialised in endometriosis.

Endometriosis symptoms typically manifest before the age of 20 and persist throughout the reproductive phase of life [4]. This phase imposes not only direct healthcare costs associated with surgeries and outpatient care but also substantial indirect costs due to fatigue and diminished productivity. These indirect costs are estimated to amount to an annual total of 22 billion USD [29]. The constant pressure to perform, heightened stress levels resulting from chronic pain, and the impact on social life due to pain, or the need for frequent restroom access significantly have a significant effect on the mental well-being of endometriosis patients [30]. These challenges are further compounded by the stress experienced by partners due to infertility and the disruption of intimate relationships caused by dyspareunia and depression. A study by Culley et al. [30] reported that 33.5–71% of affected women perceived endometriosis as having a substantial negative impact on their sex life, with a correlation between dyspareunia and a negative impact on their partnerships. Our study aligns with these findings, as 95.3% of participants reported that pelvic pain had affected their sexual behaviour in the previous month, and 97.4% of participants with dyspareunia indicated that endometriosis had strained their partnerships.

### Strengths and limitations

This study, the first of its kind in Europe, delves into the self-rated effectiveness of both pharmacological and non-pharmacological approaches for the treatment of endometriosis-associated pain. The study was conducted with a large sample size and a high response rate of 65.2% and only included participants with diagnosed endometriosis. Despite the time commitment of up to 40 min, participants may have been motivated to respond due to frustration with their pain experiences despite undergoing existing medical therapy. The study's comprehensive description of the medical condition and therapy options, along with the large number of participants, makes the results highly valuable for health and social policy in German-speaking countries.

Despite these strengths, it is essential to acknowledge certain limitations. A significant limitation of online surveys is the potential for selection bias due to the specific population that participates. To address this limitation, a sample size calculation was conducted, with the calculation based on the prevalence of endometriosis [2, 3]. This methodological approach enhances the validity of the findings and ensures that the study results are as representative as possible within the constraints of an online survey. Pain perception is inherently subjective, and self-reported measurements can introduce bias. A study conducted by Saha et al. reported the validity of self-reported data in endometriosis [31]. Moreover, although the presence of infections or malignant diseases was asked in the questionnaire, other diseases could contribute to the reported pain levels. All data were collected anonymously and without direct medical professional oversight, which permitted the disclosure of sensitive information, such as the use of illicit substances, although there was no corroboration for the reported endometriosis diagnoses.

## Conclusions

Endometriosis has a significant impact on the lives of those affected. Addressing its complexities and the lack of a cure, it is necessary to adopt a multimodal interdisciplinary approach, including medical treatment, mental health support, work-related considerations, and economic assistance [4, 32]. Since the absence of a cure for endometriosis, self-management techniques and lifestyle changes can be valuable tools for women to take control of their health and complement their existing treatment plans. Empowering women through these techniques can help them manage their condition and support their treatment regimens. It is worth noting that there is limited research on the effectiveness and mechanisms of action of specific self-management techniques for endometriosis, which poses a barrier to insurance coverage. Therefore, it is crucial to conduct further studies to assess the efficacy of non-pharmacological therapies, and for insurance companies to provide financial support to facilitate the development of improved and safer treatments for patients with endometriosis.

## Supplementary Information

Below is the link to the electronic supplementary material.

Supplementary file1 (PDF 2493 KB)

## Data Availability

The data that support the findings of this study are available from the corresponding author, SM, upon reasonable request.
