# Supplementary material for: The role of self-management in endometriosis pain: insights from a cross-sectional survey in Germany, Austria, and Switzerland
Source: Arch Gynecol Obstet. 2025 Apr 19;312(2):425–34. doi: 10.1007/s00404-025-08019-1 (PMC12334507; doi:10.1007/s00404-025-08019-1)
Supplement: Supplementary file 1 — Supplementary file1 (PDF 2493 KB) [file 404_2025_8019_MOESM1_ESM.pdf]

CannabisEM → base 07.07.2022, 13:53

Seite 01

IN01

Sehr geehrte Studieninteressierte,

Endometriose (EM) ist eine chronisch-entzündliche Erkrankung, von der ca. 10% der Frauen im reproduktionsfähig Alter betroffen sind, also etwa 2 Millionen Menschen in Deutschland und 270 Millionen weltweit. EM ist durch das Wachstum von uterusschleimhautähnlichen Zellverbänden außerhalb der Gebärmutterhöhle charakterisiert. Häufige Symptome sind wiederkehrende oder chronische Schmerzen, übermäßige Erschöpfung und Fruchtbarkeitsprobleme, welche die psychische Gesundheit, Sexualität, Arbeitsfähigkeit und allgemeine Lebensqualität der Patient*innen erheblich beeinträchtigen. Bis heute gibt es keine kausale Therapie.

Die Erkrankung Endometriose wird häufig mit Schmerzmitteln behandelt, aber auch mit nicht-pharmakologischen, also komplementären Verfahren. Wir sind an Ihren Erfahrungen zur Selbstbehandlung interessiert. Dabei interessieren uns die Einnahme von Schmerzmitteln, aber auch andere Maßnahmen wie das Erlernen von Entspannungstechniken, Yoga-Übungen, Atemtechniken oder Ernährungsumstellungen, genauso wie die Verwendung von Alkohol oder Cannabis. Wir möchten untersuchen, wie Menschen mit Endometriose versuchen, ihre Schmerzen zu lindern und welche Erfahrungen sie damit haben.
Die folgenden Informationen sollen Sie über das Forschungsprojekt informieren und Ihnen helfen, sich zu entscheiden, ob Sie daran teilnehmen möchten. Bitte nehmen Sie sich Zeit, diese Informationen aufmerksam zu lesen.

Was ist das Ziel dieses Forschungsprojektes?
In einem umfassenden und ganzheitlichen Fragebogendesign wird in diesem Projekt die Stärke von Endometriose-assoziierten Schmerzen unter Einsatz unterschiedlicher Behandlungsmaßnahmen (sowohl pharmakologisch als nicht-pharmakologisch) untersucht. Die Erhebung dieser Daten gibt uns Einblicke in Ihren Bedarf und lässt uns an Ihren Erfahrungen teilnehmen. So können wir künftige Therapiestrategien besser an Ihre Bedürfnisse anpassen.

Beruht die Teilnahme an dem Forschungsprojekt auf Freiwilligkeit?
Ihre Teilnahme an dem Forschungsprojekt beruht auf Freiwilligkeit. Falls Sie nicht teilnehmen möchten, entstehen Ihnen daraus keine Nachteile. Das Ausfüllen des Fragebogens nimmt ca. 15 bis 40 Minuten in Anspruch.

Was passiert, wenn ich an diesem Forschungsprojekt teilnehme?
Wenn Sie auf "Ja" für Ihre Einwilligung klicken, werden Sie automatisch zum Online-Fragebogen weitergeleitet.

Was sind mögliche Risiken und Vorteile, falls ich an dem Forschungsprojekt teilnehme?
Es entstehen keine Risiken für Sie durch die Teilnahme an der Studie. Die Teilnahme hat keinen Einfluss auf Ihre medizinische Behandlung. Durch die Teilnahme an dem Forschungsprojekt werden Ihnen keine Kosten entstehen.

Ihre Teilnahme kann weiterhin dazu beitragen, dass in Zukunft bessere Therapiemöglichkeiten für Menschen mit Endometriose zur Verfügung stehen.

Verarbeitung Ihrer personenbezogenen Daten
Im Rahmen unserer Umfrage erheben und verarbeiten wir personenbezogene Daten, wie z.B. Informationen über Alter, Geschlecht, Angaben zu Schulausbildung und Berufstätigkeit, Familiengröße, chronische Erkrankungen, ohne aber den Namen oder eindeutige Bezugsgrößen zu erheben, die einen unmittelbaren Bezug zu Ihrer Person ermöglichen. Die Teilnahme an dieser Umfrage erfolgt ohne die Nennung Ihres Namens oder Daten, die eine Identifikation ermöglichen. Eine Registrierung über das Online-Portal ist für die Teilnahme nicht erforderlich. Sofern es sich aufgrund der spezifischen Antworten um personenbeziehbare Daten handelt, werden wir diese vertraulich behandeln.

Grundsätzlich können aufgrund der Anonymität der Umfrage und mangels Personenbeziehbarkeit/ Rückverfolgbarkeit die Rechte auf Auskunft, Berichtigung, Löschung und Widerruf nicht geltend gemacht werden. Sollte jedoch im Einzelfall aufgrund der Beantwortung der Fragen ein Rückschluss auf Ihre Identität möglich sein, steht Ihnen das Fragerecht und das Beschwerderecht zur Verfügung.
Bitte beachten Sie, dass die Ergebnisse der Studie in der medizinischen Fachliteratur veröffentlicht werden können, wobei Ihre Identität jedoch nicht bekannt wird, weil wir die personenbeziehbaren Daten entfernen.

Die gesammelten Daten und Ergebnisse werden für einen Zeitraum von mindestens 10 Jahren vom Klinikum aufbewahrt werden. Sie dürfen von spezialisierten Dienstleistern in gesicherten Bereichen aufbewahrt bzw. gespeichert werden entsprechend den Datenschutzgesetzen. Die im Rahmen dieses Forschungsprojektes erhobenen Daten werden eventuell für zukünftige Forschungsprojekte auf dem Gebiet der Endometriose verwendet und nicht ohne Ihre gesonderte Einwilligung in ein Drittland ohne angemessenes Datenschutzniveau weitergeben. Sie können sich über Nachfragen bei der Studienleitung über die Verwendung Ihrer Daten aktuell informieren.

Im Falle von Fragen oder Anmerkungen, wenden Sie sich gerne an: die Studienleitung, die die für die Verarbeitung der personenbezogenen Daten verantwortliche Stelle darstellt:
Prof. Dr. med. Sylvia Mechsner
Abteilung für Gynäkologie
Augustenburger Platz 1, 13353 Berlin
Tel: 0049 30 450 664866
E-mail: sylvia.mechsner@charite.de

Bei Anliegen zur Datenverarbeitung und zur Einhaltung der datenschutzrechtlichen Anforderungen können Sie sich auch an die Stabsstelle Datenschutz der Charité wenden:
Stabsstelle Datenschutz
Charitéplatz 1, 10117 Berlin

Telefon: 030 450580016
E-Mail: datenschutz@charite.de

Für den Fall, dass Sie eine Datenverarbeitung für rechtswidrig halten, haben Sie neben der Inanspruchnahme gerichtlicher Hilfe die Möglichkeit, Beschwerde einzureichen bei der für die Charité Universitätsmedizin Berlin zuständigen Aufsichtsbehörde. Dies ist die
Berliner Beauftragte für Datenschutz und Informationsfreiheit
Friedrichstraße 219, 10969 Berlin.
Telefon: +49 30 13889-0
Fax: +49 30 2155050
E-Mail: mailbox@datenschutz-berlin.de

Bleibt meine Teilnahme an dem Forschungsprojekt vertraulich?
Ihre Teilnahme an dem Forschungsprojekt wird vertraulich behandelt. Im Rahmen des Forschungsprojektes werden besondere Sicherheitsmaßnahmen ergriffen, um einen unerlaubten Zugriff auf Ihre Daten zu verhindern. Ihre Daten werden nur pseudonymisiert verwendet und können so Ihrer Person nicht zugeordnet werden. Auch die im Rahmen des Forschungsprojektes gewonnenen Ergebnisse weisen keinen Bezug zu Ihrer Person auf. Die Ergebnisse der Untersuchungen werden nicht in Ihre Krankenakte am Klinikum aufgenommen und werden nicht an Ihre Ärzt*innen oder Ihre Krankenkasse weitergegeben.

Werde ich über die Ergebnisse des Forschungsprojektes informiert?
Das Forschungsprojekt dient der Evaluation von komplementären und gängigen Therapieverfahren, die von Menschen mit Endometriose angewandt werden. Dies ist ein langwieriger Prozess. Die im Rahmen des Forschungsprojektes gewonnenen Ergebnisse sind grundlegender Natur. Sie werden sehr wahrscheinlich keine Erkenntnisse liefern, die einen unmittelbaren Einfluss auf Entscheidungen über Ihre jetzige Behandlung haben. Sie werden daher nicht über die Ergebnisse informiert.

Was passiert mit den Ergebnissen des Forschungsprojektes und habe ich einen kommerziellen Vorteil?
Die Ergebnisse oder Teile davon können in wissenschaftlichen Zeitschriften, Büchern oder auf Kongressen veröffentlicht werden. Die Veröffentlichungen und Berichte werden keinen Bezug zu Ihrer Person enthalten.

Das Klinikum darf die Ergebnisse wirtschaftlich nutzen, wie z.B. für die Entwicklung von medizinischen Tests, Therapien und Diagnoseverfahren.

Aus der Bereitstellung und Verwendung Ihrer pseudonymisierten Daten bzw. aus der wirtschaftlichen Nutzung der erzielten Ergebnisse oder der entwickelten pharmazeutischen und diagnostischen Produkte entstehen für Sie keine finanziellen oder anderweitigen Ansprüche. Ihnen entstehen keine Kosten und Sie erhalten keine finanzielle Aufwandsentschädigung für Ihre Teilnahme.

Kann ich die Teilnahme an diesem Forschungsprojekt widerrufen?
Ihre Einwilligung zur Überlassung der Daten ist freiwillig. Sie können jederzeit ohne Angabe von Gründen die Teilnahme an dem Forschungsprojekt beenden. Dies hat keinerlei Auswirkung auf Ihre medizinische Behandlung. Aufgrund der Anonymität der Umfrage und mangels Personenbeziehbarkeit/Rückverfolgbarkeit können die Rechte auf Auskunft, Berichtigung, Löschung und Widerruf nicht geltend gemacht werden.

Versicherung
Für diese Studie wurde keine spezielle Versicherung für die Studienteilnehmer*innen abgeschlossen. Die an der Studie beteiligten Mitarbeiter*innen der Charité (Studienärzt*innen, Studienpfleger*innen) sind durch die Betriebshaftpflichtversicherung der Charité gegen Haftpflichtansprüche, welche aus ihrem schuldhaften Verhalten resultieren könnten, versichert.

Prof. Dr. med. Sylvia Mechsner
Studienleiterin

Prof. Dr. med. Jalid Sehouli
Leiter der Klinik für Gynäkologie

KONTAKTADRESSEN:
Studienleiterin Prof. Dr. med. Sylvia Mechsner
Abteilung für Gynäkologie, Charité-Universitätsmedizin Berlin, Campus Virchow Klinikum, Augustenburger Platz 1, 13353 Berlin, Germany
Tel: 0049 30 450664866, e-mail: sylvia.mechsner@charite.de

IN02

Einverständniserklärung

Einwilligung in die Verarbeitung meiner personenbezogenen Daten:

Mir ist bekannt, dass bei dieser Studie personenbezogene insbesondere auch sensible Daten zu Gesundheit, Geschlecht, Alter, Familienstand und Bildungsabschluss verarbeitet werden sollen. Die Verarbeitung der Daten setzt gemäß Art. 6 Abs. 1 lit. a, Art. 9 Abs. 2 lit. a der Datenschutz-Grundverordnung (DS-GVO) die Abgabe folgender Einwilligungserklärung voraus:

Ich wurde anhand des Informationsblattes ausführlich und verständlich darüber aufgeklärt, dass meine in der Studie erhobenen Daten, insbesondere Angaben über meine Gesundheit und mein Geschlecht, Alter, Familienstand und Bildungsabschluss sowie Informationen über meine Gesundheit zu den in dem Informationsblatt zur Studie beschriebenen Zwecken erhoben und in pseudonymisierter Form gespeichert und ausgewertet werden. Ich bin damit einverstanden, dass die Studienergebnisse in anonymer Form veröffentlicht werden dürfen und dass bei medizinischen Journalen die Datensätze ohne Pseudonym hinterlegt werden dürfen.

Mir ist bekannt, dass eine Rückverfolgung der Datenverarbeitung ausgeschlossen ist, sodass ich meine Rechte auf Auskunft, Berichtigung oder Löschung nicht mehr durchsetzen kann. Außerdem kann ich Beschwerde bei einer Datenschutzbehörde einlegen.

Der Anonymisierung meiner personenbezogenen Daten zum Zwecke der Veröffentlichung oder Weitergabe an Kooperationspartner stimme ich zu.

Ich wurde darüber aufgeklärt, dass ich meine Einwilligung in die Datenverarbeitung jederzeit für die Zukunft widerrufen kann und dass der Widerruf die Rechtmäßigkeit der bereits erfolgten Datenverarbeitung nicht berührt. Der Widerruf hat zudem keine Auswirkungen, sollte hierdurch die Durchführung des Forschungsvorhabens unmöglich oder ernsthaft beeinträchtigt werden. Eine Ausfertigung der Informationen und der Einwilligungserklärung kann ich jederzeit unter https://.....

- ◯ Ja, ich möchte an der Befragung teilnehmen, bin über 18 Jahre alt und bin damit einverstanden, dass meine Daten in anonymisierter Form für wissenschaftliche Zwecke verwendet werden. (leitet weiter zum Beginn des Fragebogens)
- ◯ Nein, ich möchte an der Befragung nicht teilnehmen.

1 aktive(r) Filter

Filter IN02/F1
Wenn eine der folgenden Antwortoption(en) ausgewählt wurde: **2**
Dann nach dem Klick auf "Weiter" den Text **IN03** anzeigen und das Interview beenden

Seite 02

SD02

1. Wie alt sind Sie?

Ich bin [ ] Jahre

SD07

2. In welchem Land leben Sie derzeit?

- ◯ Deutschland
- ◯ Österreich
- ◯ Schweiz

SD10

3. Welches ist der höchste Bildungsabschluss, den Sie haben?

- ◯ Noch Schülerin
- ◯ Schule beendet ohne Abschluss
- ◯ Hauptschulabschluss/Volksschulabschluss
- ◯ Realschulabschluss (Mittlere Reife)
- ◯ Abschluss Polytechnische Oberschule 10. Klasse (vor 1965: 8. Klasse)
- ◯ Fachhochschulreife (Abschluss einer Fachoberschule)
- ◯ Abitur, allgemeine oder fachgebundene Hochschulreife (Gymnasium bzw. EOS)
- ◯ Hochschulabschluss
- ◯ Anderer Schulabschluss: [ ]

SD12

4. Welchen beruflichen Bildungsabschluss haben Sie?

Bitte wählen Sie den höchsten Bildungsabschluss, den Sie bisher erreicht haben.

- ◯ Keinen beruflichen Ausbildungsabschluss
- ◯ Beruflich-betriebliche Anlernzeit mit Abschlusszeugnis, aber keine Lehre
- ◯ Teilfacharbeiterabschluss
- ◯ Abgeschlossene gewerbliche oder landwirtschaftliche Lehre
- ◯ Abgeschlossene kaufmännische Lehre
- ◯ Berufliches Praktikum, Volontariat
- ◯ Berufsfachschulabschluss
- ◯ Fachschulabschluss
- ◯ Meister-, Techniker- oder gleichwertiger Fachschulabschluss
- ◯ Fachhochschulabschluss
- ◯ Hochschulabschluss
- ◯ Anderer Abschluss, und zwar: [ ]

SD13

5. Sind Sie momentan erwerbstätig?

- ◯ Ja, ich bin erwerbstätig.
- ◯ Nein, ich bin arbeitslos.
- ◯ Nein, ich bin Rentnerin.
- ◯ Nein, ich bin Hausfrau.
- ◯ Nein, ich bin nichts von alledem.

SD16

6. Wie hoch ist ungefähr Ihr monatliches Nettoeinkommen?

Gemeint ist der Betrag, der sich aus allen Einkünften zusammensetzt und nach Abzug der Steuern und Sozialversicherungen übrig bleibt.

- ◯ Ich habe kein eigenes Einkommen
- ◯ unter 500 €
- ◯ 500 € bis unter 1000 €
- ◯ 1000 € bis unter 1500 €
- ◯ 1500 € bis unter 2000 €
- ◯ 2000 € bis unter 2500 €
- ◯ 2500 € bis unter 3000 €
- ◯ 3000 € oder mehr

SD19

7. Wie ist Ihr aktueller Familienstand? (Mehrfachantwort möglich)

- ☐ ledig, keine Beziehung
- ☐ alleinerziehend
- ☐ verheiratet bzw. eingetragene Partnerschaft
- ☐ in einer festen Partnerschaft
- ☐ geschieden
- ☐ Sonstiges

SD18

8. Haben Sie das Gefühl, dass Ihre Endometriose Ihre Partnerschaft belastet?

Hinweis: Belastungen durch die Endometriose können beispielsweise durch Schmerzen im Allgemeinen, Schmerzen beim Geschlechtsverkehr, verminderte Libido oder Lubrikation durch eine Hormonbehandlung oder Fertilitätsbeeinträchtigungen bestehen.

◯	◯	◯	◯	◯	◯
0 gar nicht von der Endometriose belastet	1	2	3	4	5 extrem von der Endometriose belastet

SD20

9. Haben Sie das Gefühl, dass Ihre Endometriose Ihre letzte Partnerschaft belastet hat?

Hinweis: Belastungen durch die Endometriose können beispielsweise durch Schmerzen im Allgemeinen, Schmerzen beim Geschlechtsverkehr, verminderte Libido oder Lubrikation, eine Hormonbehandlung oder Fertilitätsbeeinträchtigungen bestehen.

○	○	○	○	○	○
0 gar nicht von der Endometriose belastet	1	2	3	4	5 extrem von der Endometriose belastet

SD17

10. Haben Sie eine bösartige Erkrankung oder eine Infektion? (Bsp.: Krebserkrankung, Tuberkulose, Hepatitis A/B/C)

- ○ Nein
- ○ Ja

SD21

11. Rauchen Sie Zigaretten?

- ○ Nein
- ○ Ja

12. Wann wurde bei Ihnen Endometriose diagnostiziert? EM01

(bitte Monat und Jahr angeben)

13. Wann traten bei Ihnen zum ersten Mal endometriosetypische Beschwerden auf? EM02

bitte Jahr angeben

14. Wie wurde bei Ihnen Endometriose diagnostiziert? EM03

- ◯ Bauchspiegelung/Laparoskopie/operativer Eingriff
- ◯ MRT
- ◯ Ultraschall
- ◯ Sonstiges

15. Sind Sie schon einmal wegen Endometriose oder Verdacht auf Endometriose operiert worden? EM04

- ◯ Nein
- ◯ Ja

8 aktive(r) Filter

Filter EM04/F1
Wenn eine der folgenden Antwortoption(en) ausgewählt wurde: **2**
Dann Seite(n) **OP** des Fragebogens anzeigen (sonst ausblenden)

Filter EM04/F2
Wenn eine der folgenden Antwortoption(en) ausgewählt wurde: **2**
Dann Seite(n) **OP** des Fragebogens anzeigen (sonst ausblenden)

Filter EM04/F3
Wenn eine der folgenden Antwortoption(en) ausgewählt wurde: **1**
Dann Frage/Text **EM05** später im Fragebogen ausblenden

Filter EM04/F4
Wenn eine der folgenden Antwortoption(en) ausgewählt wurde: **1**
Dann Frage/Text **EM06** später im Fragebogen ausblenden

Filter EM04/F5
Wenn eine der folgenden Antwortoption(en) ausgewählt wurde: **-9**
Dann Frage/Text **EM05** später im Fragebogen ausblenden

Filter EM04/F6
Wenn eine der folgenden Antwortoption(en) ausgewählt wurde: **-9**
Dann Frage/Text **EM06** später im Fragebogen ausblenden

Filter EM04/F7
Wenn eine der folgenden Antwortoption(en) ausgewählt wurde: **2**
Dann Frage/Text **EM17** später im Fragebogen anzeigen (sonst ausblenden)

Filter EM04/F8
Wenn eine der folgenden Antwortoption(en) ausgewählt wurde: **2**
Dann Frage/Text **EM18** später im Fragebogen anzeigen (sonst ausblenden)

16. Wie viele Operationen hatten Sie bereits insgesamt aufgrund Ihrer Endometriose? EM05

- ◯ 1 Operation
- ◯ 2 Operationen
- ◯ 3 Operationen
- ◯ 4 Operationen
- ◯ 5 Operationen
- ◯ mehr als 5 Operationen

17. Wurde die Operation in einem zertifizierten Endometriosezentrum durchgeführt? EM06

- ◯ Nein
- ◯ Ja
- ◯ Ich weiß es nicht

18. Welches ASF/ rASRM-Stadium ist angegeben? EM17
(Mehrfachantwort möglich)

☐ Keine Angabe ☐ I ☐ II ☐ III ☐ IV

19. Was ist nach ENZIAN-Klassifikation angegeben? EM18
(Mehrfachantwort möglich)

☐ A1	☐ A2	☐ A3	☐ FA	☐ FI
☐ B1	☐ B2	☐ B3	☐ FB	☐ FO
☐ C1	☐ C2	☐ C3	☐ FU	☐ keine Angabe

EM07

20. Wie viele verschiedene Hormonpräparate haben Sie schon zur Behandlung der Endometriose angewendet? (Inkl. aktuellem Präparat)

- ◯ 0 Präparate
- ◯ 1 Präparat
- ◯ 2 Präparate
- ◯ 3 Präparate
- ◯ 4 Präparate
- ◯ 5 Präparate
- ◯ mehr als 5 Präparate

3 aktive(r) Filter

Filter EM07/F1
Wenn eine der folgenden Antwortoption(en) ausgewählt wurde: **7**
Dann Frage/Text **EM19** später im Fragebogen ausblenden

Filter EM07/F2
Wenn eine der folgenden Antwortoption(en) ausgewählt wurde: **7**
Dann Frage/Text **EM08** später im Fragebogen ausblenden

Filter EM07/F3
Wenn eine der folgenden Antwortoption(en) ausgewählt wurde: **7**
Dann Frage/Text **EM09** später im Fragebogen ausblenden

EM20

21. Sind Sie Blutungsfrei?

Gemeint ist, ob die Einnahme von aktuellen Medikamenten dazu führt, dass Ihre Regelblutung unterbunden wird.

- ◯ Ja
- ◯ Nein

Seite 06
PA

EM10

22. Haben Sie Schmerzen wegen der Endometriose?

Zyklusabhängige Unterbauchschmerzen	Nein	Ja
Zyklusunabhängige Unterbauchschmerzen	Nein	Ja
Schmerzhafte Menstruation, typischerweise mit Bauchkrämpfen	Nein	Ja
Schwieriger oder schmerzhafter Geschlechtsverkehr	Nein	Ja
Schmerzen oder Schwierigkeiten beim Stuhlgang	Nein	Ja
Schmerzhaftes Wasserlassen als Unbehagen oder Brennen beim Wasserlassen	Nein	Ja

EM11

23. Welche Medikamente haben Sie bisher gegen Endometriose-assoziierte Schmerzen eingenommen?

Diclofenac	Niemals	Ja, ich habe schon eingenommen (Vergangenheit)	Ja, ich nehme jetzt (heutzutage)
Buscopan	Niemals	Ja, ich habe schon eingenommen (Vergangenheit)	Ja, ich nehme jetzt (heutzutage)
Actylsalicylsäure (ASS)	Niemals	Ja, ich habe schon eingenommen (Vergangenheit)	Ja, ich nehme jetzt (heutzutage)
Celecoxib	Niemals	Ja, ich habe schon eingenommen (Vergangenheit)	Ja, ich nehme jetzt (heutzutage)
Ibuprofen	Niemals	Ja, ich habe schon eingenommen (Vergangenheit)	Ja, ich nehme jetzt (heutzutage)
Paracetamol	Niemals	Ja, ich habe schon eingenommen (Vergangenheit)	Ja, ich nehme jetzt (heutzutage)
Metamizol	Niemals	Ja, ich habe schon eingenommen (Vergangenheit)	Ja, ich nehme jetzt (heutzutage)
Tramadol	Niemals	Ja, ich habe schon eingenommen (Vergangenheit)	Ja, ich nehme jetzt (heutzutage)
Tilidin	Niemals	Ja, ich habe schon eingenommen (Vergangenheit)	Ja, ich nehme jetzt (heutzutage)
Dihydrocodein	Niemals	Ja, ich habe schon eingenommen (Vergangenheit)	Ja, ich nehme jetzt (heutzutage)
Morphin	Niemals	Ja, ich habe schon eingenommen (Vergangenheit)	Ja, ich nehme jetzt (heutzutage)
Oxycodon	Niemals	Ja, ich habe schon eingenommen (Vergangenheit)	Ja, ich nehme jetzt (heutzutage)
Levomethadon	Niemals	Ja, ich habe schon eingenommen (Vergangenheit)	Ja, ich nehme jetzt (heutzutage)

Fentanyl	Niemals	Ja, ich habe schon eingenommen (Vergangenheit)	Ja, ich nehme jetzt (heutzutage)
Pethidin	Niemals	Ja, ich habe schon eingenommen (Vergangenheit)	Ja, ich nehme jetzt (heutzutage)
Buprenorphin	Niemals	Ja, ich habe schon eingenommen (Vergangenheit)	Ja, ich nehme jetzt (heutzutage)
Piritramid	Niemals	Ja, ich habe schon eingenommen (Vergangenheit)	Ja, ich nehme jetzt (heutzutage)
Koanalgetika (z.B Antidepressiva, Muskelrelaxanzien)	Niemals	Ja, ich habe schon eingenommen (Vergangenheit)	Ja, ich nehme jetzt (heutzutage)
Sonstiges	Niemals	Ja, ich habe schon eingenommen (Vergangenheit)	Ja, ich nehme jetzt (heutzutage)

EM13

24. Wie wurden die Medikamente beschafft?

(Mehrfachnennung möglich)

- ☐ Selbstbeschaffung
- ☐ Rezept von der/dem Frauenärzt*in
- ☐ Rezept von der/dem Schmerzen Therapeut*in
- ☐ Rezept von der/dem Hausärzt*in

EM15

25. Wie ist/war die typische Schmerzstärke unter der Schmerzmedikation?

0 kein Schmerz
1
2
3
4
5
6
7
8
9
10 stärkster vorstellbarer Schmerz

EM16

26. Auf einer Skala von 0-10, wie effektiv würden Sie das Medikament für die Reduktion Ihrer endometriosebedingten Unterleibsschmerzen beurteilen?

- 0 überhaupt nicht effektiv
- 1
- 2
- 3
- 4
- 5
- 6
- 7
- 8
- 9
- 10 sehr effektiv

EM19

27. Wie heißt Ihr aktuelles Hormonpräparat?

Wenn Sie aktuell keine Hormone einnehmen, schreiben Sie bitte „Keine".

EM08

28. Wie gut vertragen Sie Ihr aktuelles Hormonpräparat?

○ sehr schlecht ○ mittel ○ sehr gut ○ keine aktuelle Hormoneinnahme

EM21

29. Wie ist/war die typische Schmerzstärke unter der Hormontherapie?

0 kein Schmerz
1
2
3
4
5
6
7
8
9
10 stärkster vorstellbarer Schmerz

EM09

30. Auf einer Skala von 0-10, wie effektiv würden Sie das Hormonpräparat für die Reduktion Ihrer endometriosebedingten Unterleibsschmerzen beurteilen?

0 überhaupt nicht effektiv
1
2
3
4
5
6
7
8
9
10 sehr effektiv

Seite 08
SC

SC01

31. Wie sehr haben Ihre Unterleibsschmerzen im letzten Monat folgende Aspekte beeinflusst?

	gar nicht	kaum	mäßig	sehr	extrem
Leistung	○	○	○	○	○
Stimmung	○	○	○	○	○
Schlaf	○	○	○	○	○
Verdauung	○	○	○	○	○
Sitzen (länger als 20 Minuten)	○	○	○	○	○
Alltag zu Hause/ in der Schule/ Uni/ auf der Arbeit	○	○	○	○	○
körperliche Aktivitäten (z.B. Joggen, Yoga, Fahrradfahren)	○	○	○	○	○
Tragen bestimmter Kleidungsstücke (z.B. enge Kleidung/ Unterwäsche)	○	○	○	○	○
Sexualverhalten (z.B. Masturbation, Geschlechtsverkehr)	○	○	○	○	○

SC03

32. Haben Sie in den letzten 6 Monaten nicht-pharmakologische Therapieoptionen (wie z.B. Yoga, Meditation/ Entspannungstechniken, Ernährungsumstellung, Wärmeanwendungen, Hanföl) genutzt, um besser mit Ihren Endometriosebeschwerden zurecht zu kommen oder zur Reduktion der Nebenwirkungen von eingenommenen Medikamenten (z.B. Schmerzmedikamente) zur Behandlung der Endometriose?

○ Nein
○ Ja

5 aktive(r) Filter

Filter SC03/F1
Wenn eine der folgenden Antwortoption(en) ausgewählt wurde: **2**
Dann Frage/Text **SC05** später im Fragebogen anzeigen (sonst ausblenden)

Filter SC03/F2
Wenn eine der folgenden Antwortoption(en) ausgewählt wurde: **1**
Dann Frage/Text **SC05** später im Fragebogen ausblenden

Filter SC03/F3
Wenn eine der folgenden Antwortoption(en) ausgewählt wurde: **1**
Dann Frage/Text **SC04** später im Fragebogen anzeigen (sonst ausblenden)

Filter SC03/F4
Wenn eine der folgenden Antwortoption(en) ausgewählt wurde: **2**
Dann Frage/Text **SC04** später im Fragebogen ausblenden

Filter SC03/F5
Wenn eine der folgenden Antwortoption(en) ausgewählt wurde: **1**
Dann Seite(n) **YO-CA, OS, TC** des Fragebogens ausblenden

33. Aus welchen Gründen nutzen Sie solche Therapieoptionen nicht? SC04

bitte alle zutreffenden Gründe auswählen, wenn Sie keine solcher nicht-pharmakologischen Maßnahmen nutzen

- ☐ Kosten
- ☐ Zeitaufwand
- ☐ bereits ausprobiert und haben nicht geholfen
- ☐ zu wenig Informationen dazu
- ☐ schwere Zugangsmöglichkeiten
- ☐ weitere

SC05

34. Welche von den folgenden nicht-pharmakologischen Maßnahmen zur Bewältigung Ihrer Endometriosebeschwerden haben Sie in den letzten 6 Monaten genutzt?

bitte alle zutreffenden Optionen auswählen

- ☐ Yoga/ Pilates
- ☐ Tai Chi/ Qi Gong
- ☐ Meditation/ Atemübungen
- ☐ Ausruhen/ Erholen
- ☐ Dehnungsübungen
- ☐ sportliche körperliche Aktivitäten (z.B. Spazieren gehen, Joggen, Schwimmen, Krafttraining)
- ☐ Wärme (z.B. Wärmekissen, Sauna, warmes Bad)
- ☐ Kälte (z.B. Kältekompresse, kalt duschen)
- ☐ Akkupunktur
- ☐ Osteopathie
- ☐ Massage
- ☐ Traditionelle chinesische Medizin (TCM)
- ☐ Alkoholkonsum
- ☐ pflanzliche Beruhigungs-/ Entspannungsmittel (z.B. Substanzen mit Kamille, Kava, Passionsblume)
- ☐ bestimmte Ernährungsweisen (z.B. Paleo-Diät, pflanzenbasierte Kost, FODMAP-Diät)
- ☐ Hanf-/ CBD-Öl
- ☐ Cannabis oder cannabishaltige Produkte (verschrieben oder illegal)
- ☐ Sonstiges

17 aktive(r) Filter

Filter SC05/F1
Wenn eine der folgenden Antwortoption(en) ausgewählt wurde: **1**
Dann Seite(n) **YO** des Fragebogens anzeigen (sonst ausblenden)

Filter SC05/F2
Wenn eine der folgenden Antwortoption(en) ausgewählt wurde: **2**
Dann Seite(n) **TA** des Fragebogens anzeigen (sonst ausblenden)

Filter SC05/F3
Wenn eine der folgenden Antwortoption(en) ausgewählt wurde: **3**
Dann Seite(n) **ME** des Fragebogens anzeigen (sonst ausblenden)

Filter SC05/F4
Wenn eine der folgenden Antwortoption(en) ausgewählt wurde: **4**
Dann Seite(n) **AU** des Fragebogens anzeigen (sonst ausblenden)

Filter SC05/F5
Wenn eine der folgenden Antwortoption(en) ausgewählt wurde: **5**
Dann Seite(n) **DE** des Fragebogens anzeigen (sonst ausblenden)

Filter SC05/F6
Wenn eine der folgenden Antwortoption(en) ausgewählt wurde: **6**
Dann Seite(n) **SP** des Fragebogens anzeigen (sonst ausblenden)

Filter SC05/F7
Wenn eine der folgenden Antwortoption(en) ausgewählt wurde: **7**
Dann Seite(n) **WA** des Fragebogens anzeigen (sonst ausblenden)

Filter SC05/F8
Wenn eine der folgenden Antwortoption(en) ausgewählt wurde: **8**
Dann Seite(n) **KA** des Fragebogens anzeigen (sonst ausblenden)

Filter SC05/F9
Wenn eine der folgenden Antwortoption(en) ausgewählt wurde: **9**
Dann Seite(n) **AK** des Fragebogens anzeigen (sonst ausblenden)

Filter SC05/F10
Wenn eine der folgenden Antwortoption(en) ausgewählt wurde: **10**
Dann Seite(n) **MA** des Fragebogens anzeigen (sonst ausblenden)

Filter SC05/F11

Wenn eine der folgenden Antwortoption(en) ausgewählt wurde: **11**
Dann Seite(n) **AL** des Fragebogens anzeigen (sonst ausblenden)

Filter SC05/F12
Wenn eine der folgenden Antwortoption(en) ausgewählt wurde: **12**
Dann Seite(n) **NA** des Fragebogens anzeigen (sonst ausblenden)

Filter SC05/F13
Wenn eine der folgenden Antwortoption(en) ausgewählt wurde: **13**
Dann Seite(n) **ER** des Fragebogens anzeigen (sonst ausblenden)

Filter SC05/F14
Wenn eine der folgenden Antwortoption(en) ausgewählt wurde: **14**
Dann Seite(n) **OL** des Fragebogens anzeigen (sonst ausblenden)

Filter SC05/F15
Wenn eine der folgenden Antwortoption(en) ausgewählt wurde: **15**
Dann Seite(n) **CA** des Fragebogens anzeigen (sonst ausblenden)

Filter SC05/F16
Wenn eine der folgenden Antwortoption(en) ausgewählt wurde: **17**
Dann Seite(n) **OS** des Fragebogens anzeigen (sonst ausblenden)

Filter SC05/F17
Wenn eine der folgenden Antwortoption(en) ausgewählt wurde: **18**
Dann Seite(n) **TC** des Fragebogens anzeigen (sonst ausblenden)

Seite 11
YO

YO01

35. Wie häufig betreiben Sie Yoga durchschnittlich?

- ○ (mehrmals) täglich
- ○ 2-6 mal pro Woche
- ○ einmal wöchentlich
- ○ weniger als einmal wöchentlich

YO10

36. Wie ist/war die typische Schmerzstärke unter Yoga/Pilates?

0 kein Schmerz
1
2
3
4
5
6
7
8
9
10 stärkster vorstellbarer Schmerz

YO02

37. Auf einer Skala von 0-10, wie effektiv würden Sie das Durchführen von Yoga oder Pilates für die Reduktion Ihrer endometriosebedingten Unterleibsschmerzen beurteilen?

0 überhaupt nicht effektiv
1
2
3
4
5
6
7
8
9
10 sehr effektiv

YO03

38. Welche und wie effektiv konnten Ihre endometriosebedingten Beschwerden oder Nebenwirkungen von Medikamenten, die Sie aufgrund ihrer Endometriose einnehmen, durch Yoga oder Pilates in ihrer Intensität oder Dauer reduziert werden?

	starke Verbesserung (über 50%)	mäßige Verbesserung (25-50%)	geringe Verbesserung (unter 25%)	keine Verbesserung	Verschlechterung der Beschwerden	Ich habe dieses Symptom nicht
Übelkeit/ Erbrechen	○	○	○	○	○	○
Magen-/ Verdauungsbeschwerden	○	○	○	○	○	○
Müdigkeit	○	○	○	○	○	○
Angstzustände	○	○	○	○	○	○
Depression/ gedrückte Stimmung	○	○	○	○	○	○
Schlaf	○	○	○	○	○	○
Regelschmerzen/ zyklische Unterbauchschmerzen	○	○	○	○	○	○
azyklische Unterbauchschmerzen	○	○	○	○	○	○
Schmerzen beim Geschlechtsverkehr	○	○	○	○	○	○
Schmerzen beim Urin lassen	○	○	○	○	○	○
Schmerzen beim Stuhlgang	○	○	○	○	○	○

YO04

39. Haben Sie eine andere Wirkung von der Durchführung von Yoga oder Pilates erwartet?

○ Ja

○ Nein

1 aktive(r) Filter

Filter YO04/F1
Wenn eine der folgenden Antwortoption(en) ausgewählt wurde: **1, 2**
Dann Frage/Text **YO05** später im Fragebogen anzeigen (sonst ausblenden)

YO05

40. Welche Erwartungen hatten Sie bezüglich der Durchführung von Yoga oder Pilates?

YO06

41. Konnte die Durchführung von Yoga oder Pilates die Menge an Medikamenten, die Sie für die Therapie ihrer endometriosebedingten Symptome normalerweise benötigen reduzieren? (z.B. die Menge an Schmerzmittel)

○ Ja, starke Reduktion (über 50%)

○ Ja, mäßige Reduktion (25-50%)

○ Ja, geringe Reduktion (unter 25%)

○ Nein

42. Wie viel hat Sie durchschnittlich die Durchführung von Yoga oder Pilates monatlich gekostet? (z.B. monatliche Mitgliedsgebühr, Anfängerkurse) YO07

43. Würden Sie Yoga/ Pilates einer Freundin oder einem Familienmitglied mit Endometriose weiterempfe[illegible]? YO08

○ Ja ○ Nein

44. Gibt es etwas weiteres, was Sie uns bezüglich Yoga/ Pilates mitteilen möchten? YO09

TA01

45. Wie häufig betreiben Sie Tai Chi/ Qi Gong durchschnittlich?

○ (mehrmals) täglich
○ 2-6 mal pro Woche
○ einmal wöchentlich
○ weniger als einmal wöchentlich

TA11

46. Wie ist/war die typische Schmerzstärke unter Tai Chi/ Qi Gong?

0 kein Schmerz
1
2
3
4
5
6
7
8
9
10 stärkster vorstellbarer Schmerz

TA10

47. Auf einer Skala von 0-10, wie effektiv würden Sie das Durchführen von Tai Chi/ Qi Gong für die Reduktion Ihrer endometriosebedingten Unterleibsschmerzen beurteilen?

0 überhaupt nicht effektiv
1
2
3
4
5
6
7
8
9
10 sehr effektiv

TA03

48. Welche und wie effektiv konnten Ihre endometriosebedingten Beschwerden oder Nebenwirkungen von Medikamenten, die Sie aufgrund ihrer Endometriose einnehmen, durch Tai Chi/ Qi Gong in ihrer Intensität oder Dauer reduziert werden?

	starke Verbesserung (über 50%)	mäßige Verbesserung (25-50%)	geringe Verbesserung (unter 25%)	keine Verbesserung	Verschlechterung der Beschwerden	Ich habe dieses Symptom nicht
Übelkeit/ Erbrechen	○	○	○	○	○	○
Magen-/ Verdauungsbeschwerden	○	○	○	○	○	○
Müdigkeit	○	○	○	○	○	○
Angstzustände	○	○	○	○	○	○
Depression/ gedrückte Stimmung	○	○	○	○	○	○
Schlaf	○	○	○	○	○	○
Regelschmerzen/ zyklische Unterbauchschmerzen	○	○	○	○	○	○
azyklische Unterbauchschmerzen	○	○	○	○	○	○
Schmerzen beim Geschlechtsverkehr	○	○	○	○	○	○
Schmerzen beim Urin lassen	○	○	○	○	○	○
Schmerzen beim Stuhlgang	○	○	○	○	○	○

TA04

49. Haben Sie eine andere Wirkung von der Durchführung von Tai Chi/ Qi Gong erwartet?

○ Ja

○ Nein

1 aktive(r) Filter

Filter TA04/F1
Wenn eine der folgenden Antwortoption(en) ausgewählt wurde: **1**
Dann Frage/Text **TA05** später im Fragebogen anzeigen (sonst ausblenden)

TA05

50. Welche Erwartungen hatten Sie bezüglich der Durchführung von Tai Chi/ Qi Gong?

TA06

51. Konnte die Durchführung von Tai Chi/ Qi Gong die Menge an Medikamenten, die Sie für die Therapie ihrer endometriosebedingten Symptome normalerweise benötigen reduzieren? (z.B. die Menge an Schmerzmittel)

○ Ja, starke Reduktion (über 50%)

○ Ja, mäßige Reduktion (25-50%)

○ Ja, geringe Reduktion (unter 25%)

○ Nein

52. Wie viel hat Sie durchschnittlich die Durchführung von Tai Chi/ Qi Gong monatlich gekostet? TA07

(z.B. monatliche Mitgliedsgebühr, Anfängerkurse)

53. Gibt es etwas weiteres, was Sie uns bezüglich Tai Chi/ Qi Gong mitteilen möchten? TA09

54. Würden Sie Tai Chi/ Qi Gong einer Freundin oder einem Familienmitglied mit Endometriose weiterempfehlen? TA08

○ Ja ○ Nein

SP01

55. Wie häufig betreiben Sie Sport/ körperliche Aktivität durchschnittlich?

○	○	○	○
(mehrmals) täglich	2-6 mal pro Woche	einmal wöchentlich	weniger als einmal wöchentlich

SP11

56. Wie ist/war die typische Schmerzstärke unter Sport/körperliche Aktivität?

0 kein Schmerz
1
2
3
4
5
6
7
8
9
10 stärkster vorstellbarer Schmerz

SP10

57. Auf einer Skala von 0-10, wie effektiv würden Sie das Durchführen von Sport/ körperliche Aktivität für die Reduktion Ihrer endometriosebedingten Unterleibsschmerzen beurteilen?

0 überhaupt nicht effektiv
1
2
3
4
5
6
7
8
9
10 sehr effektiv

SP03

58. Welche und wie effektiv konnten Ihre endometriosebedingten Beschwerden oder Nebenwirkungen von Medikamenten, die Sie aufgrund ihrer Endometriose einnehmen durch Sport/ körperliche Aktivität in ihrer Intensität oder Dauer reduziert werden?

	starke Verbesserung (über 50%)	mäßige Verbesserung (25-50%)	geringe Verbesserung (unter 25%)	keine Verbesserung	Verschlechterung der Beschwerden	Ich habe dieses Symptom nicht
Übelkeit/ Erbrechen	○	○	○	○	○	○
Magen-/ Verdauungsbeschwerden	○	○	○	○	○	○
Müdigkeit	○	○	○	○	○	○
Angstzustände	○	○	○	○	○	○
Depression/ gedrückte Stimmung	○	○	○	○	○	○
Schlaf	○	○	○	○	○	○
Regelschmerzen/ zyklische Unterbauchschmerzen	○	○	○	○	○	○
azyklische Unterbauchschmerzen	○	○	○	○	○	○
Schmerzen beim Geschlechtsverkehr	○	○	○	○	○	○
Schmerzen beim Urin lassen	○	○	○	○	○	○
Schmerzen beim Stuhlgang	○	○	○	○	○	○

SP04

59. Haben Sie eine andere Wirkung von der Durchführung von Sport/ körperlicher Aktivität erwartet?

○ Ja

○ Nein

1 aktive(r) Filter

Filter SP04/F1
Wenn eine der folgenden Antwortoption(en) ausgewählt wurde: **1**
Dann Frage/Text **SP05** später im Fragebogen anzeigen (sonst ausblenden)

SP05

60. Welche Erwartungen hatten Sie bezüglich der Durchführung von Sport/ körperlicher Aktivität?

SP06

61. Konnte die Durchführung von Sport/ körperlicher Aktivität die Menge an Medikamenten, die Sie für die Therapie Ihrer endometriosebedingten Symptome normalerweise benötigen reduzieren? (z.B. die Menge an Schmerzmittel)

- ○ Ja, starke Reduktion (über 50%)
- ○ Ja, mäßige Reduktion (25-50%)
- ○ Ja, geringe Reduktion (unter 25%)
- ○ Nein

SP07

62. Wie viel hat Sie durchschnittlich die Durchführung von Sport/ körperlicher Aktivität monatlich gekostet? (z.B. monatliche Mitgliedsgebühr, Anfängerkurse)

SP08

63. Würden Sie Sport/ körperliche Aktivität einer Freundin oder einem Familienmitglied mit Endometriose weiterempfehlen?

- ○ Ja
- ○ Nein

SP09

64. Gibt es etwas weiteres, was Sie uns bezüglich Sport/ körperliche Aktivität mitteilen möchten?

ME01

65. Wie häufig betreiben Sie Meditation/ Atemübungen durchschnittlich?

○ (mehrmals) täglich

○ 2-6 mal pro Woche

○ einmal wöchentlich

○ weniger als einmal wöchentlich

ME11

66. Wie ist/war die typische Schmerzstärke unter Meditation/Atemübungen?

0 kein Schmerz
1
2
3
4
5
6
7
8
9
10 stärkster vorstellbarer Schmerz

ME10

67. Auf einer Skala von 0-10, wie effektiv würden Sie das Durchführen von Meditation/ Atemübungen für die Reduktion Ihrer endometriosebedingten Unterleibsschmerzen beurteilen?

0 überhaupt nicht effektiv
1
2
3
4
5
6
7
8
9
10 sehr effektiv

ME03

68. Welche und wie effektiv konnten Ihre endometriosebedingten Beschwerden oder Nebenwirkungen von Medikamenten, die Sie aufgrund ihrer Endometriose einnehmen, durch Meditation/ Atemübungen in ihrer Intensität oder Dauerreduziert werden?

	starke Verbesserung (über 50%)	mäßige Verbesserung (25-50%)	geringe Verbesserung (unter 25%)	keine Verbesserung	Verschlechterung der Beschwerden	Ich habe dieses Symptom nicht
Übelkeit/ Erbrechen	○	○	○	○	○	○
Magen-/ Verdauungsbeschwerden	○	○	○	○	○	○
Müdigkeit	○	○	○	○	○	○
Angstzustände	○	○	○	○	○	○
Depression/ gedrückte Stimmung	○	○	○	○	○	○
Schlaf	○	○	○	○	○	○
Regelschmerzen/ zyklische Unterbauchschmerzen	○	○	○	○	○	○
azyklische Unterbauchschmerzen	○	○	○	○	○	○
Schmerzen beim Geschlechtsverkehr	○	○	○	○	○	○
Schmerzen beim Urin lassen	○	○	○	○	○	○
Schmerzen beim Stuhlgang	○	○	○	○	○	○

ME04

69. Haben Sie eine andere Wirkung von der Durchführung von Meditation/ Atemübungen erwartet?

○ Ja

○ Nein

1 aktive(r) Filter

Filter ME04/F1
Wenn eine der folgenden Antwortoption(en) ausgewählt wurde: **1**
Dann Frage/Text **ME05** später im Fragebogen anzeigen (sonst ausblenden)

ME05

70. Welche Erwartungen hatten Sie bezüglich der Durchführung von Meditation/ Atemübungen?

ME06

71. Konnte die Durchführung von Meditation/ Atemübungen die Menge an Medikamenten, die Sie für die Therapie Ihrer endometriosebedingten Symptome normalerweise benötigen reduzieren? (z.B. die Menge an Schmerzmittel)

○	○	○	○
Ja, starke Reduktion (über 50%)	Ja, mäßige Reduktion (25-50%)	Ja, geringe Reduktion (unter 25%)	Nein

ME07

72. Wie viel hat Sie durchschnittlich die Durchführung von Meditation/ Atemübungen monatlich gekostet? (z.B. monatliche Mitgliedsgebühr, Anfängerkurse)

ME08

73. Würden Sie Meditation/ Atemübungen einer Freundin oder einem Familienmitglied mit Endometriose weiterempfehlen?

○	○
Ja	Nein

ME09

74. Gibt es etwas weiteres, was Sie uns bezüglich Meditation/ Atemübungen mitteilen möchten?

AU01

75. Wie häufig haben Sie sich aufgrund Ihrer Endometriose durchschnittlich ausgeruht?

○ (mehrmals) täglich ○ 2-6 mal pro Woche ○ einmal wöchentlich ○ weniger als einmal wöchentlich

AU11

76. Wie ist/war die typische Schmerzstärke unter Ausruhen/Entspannen?

0 kein Schmerz
1
2
3
4
5
6
7
8
9
10 stärkster vorstellbarer Schmerz

AU10

77. Auf einer Skala von 0-10, wie effektiv würden Sie das Ausruhen/Entspannen für die Reduktion Ihrer endometriosebedingten Unterleibsschmerzen beurteilen?

0 überhaupt nicht effektiv
1
2
3
4
5
6
7
8
9
10 sehr effektiv

AU03

78. Welche und wie effektiv konnten Ihre endometriosebedingten Beschwerden oder Nebenwirkungen von Medikamenten, die Sie aufgrund ihrer Endometriose einnehmen, durch Ausruhen/Entspannen in ihrer Intensität oder Dauer reduziert werden?

	starke Verbesserung (über 50%)	mäßige Verbesserung (25-50%)	geringe Verbesserung (unter 25%)	keine Verbesserung	Verschlechterung der Beschwerden	Ich habe dieses Symptom nicht
Übelkeit/ Erbrechen	○	○	○	○	○	○
Magen-/ Verdauungsbeschwerden	○	○	○	○	○	○
Müdigkeit	○	○	○	○	○	○
Angstzustände	○	○	○	○	○	○
Depression/ gedrückte Stimmung	○	○	○	○	○	○
Schlaf	○	○	○	○	○	○
Regelschmerzen/ zyklische Unterbauchschmerzen	○	○	○	○	○	○
azyklische Unterbauchschmerzen	○	○	○	○	○	○
Schmerzen beim Geschlechtsverkehr	○	○	○	○	○	○
Schmerzen beim Urin lassen	○	○	○	○	○	○
Schmerzen beim Stuhlgang	○	○	○	○	○	○

AU04

79. Haben Sie eine andere Wirkung von dem Ausruhen/Entspannen erwartet?

○ Ja

○ Nein

1 aktive(r) Filter

Filter AU04/F1
Wenn eine der folgenden Antwortoption(en) ausgewählt wurde: **1**
Dann Frage/Text **AU05** später im Fragebogen anzeigen (sonst ausblenden)

AU05

80. Welche Erwartungen hatten Sie bezüglich des Ausruhens/Entspannen?

AU06

81. Konnte das Ausruhen/Entspannen die Menge an Medikamenten, die Sie für die Therapie Ihrer endometriosebedingten Symptome normalerweise benötigen reduzieren? (z.B. die Menge an Schmerzmittel)

○ Ja, starke Reduktion (über 50%)

○ Ja, mäßige Reduktion (25-50%)

○ Ja, geringe Reduktion (unter 25%)

○ Nein

82. Wie viel hat Sie durchschnittlich das Ausruhen/Entspannen monatlich gekostet? AU07

83. Würden Sie das Ausruhen/Entspannen einer Freundin oder einem Familienmitglied mit Endometriose weiterempfehlen? AU08

○ Ja ○ Nein

84. Gibt es etwas weiteres, was Sie uns bezüglich des Ausruhens/Entspannens mitteilen möchten? AU09

DE01

85. Wie häufig führen Sie durchschnittlich Dehnungsübungen durch?

- ○ (mehrmals) täglich
- ○ 2-6 mal pro Woche
- ○ einmal wöchentlich
- ○ weniger als einmal wöchentlich

DE11

86. Wie ist/war die typische Schmerzstärke unter Dehnungsübungen?

- 0 kein Schmerz
- 1
- 2
- 3
- 4
- 5
- 6
- 7
- 8
- 9
- 10 stärkster vorstellbarer Schmerz

DE10

87. Auf einer Skala von 0-10, wie effektiv würden Sie das Durchführen von Dehnungsübungen für die Reduktion Ihrer endometriosebedingten Unterleibsschmerzen beurteilen?

- 0 überhaupt nicht effektiv
- 1
- 2
- 3
- 4
- 5
- 6
- 7
- 8
- 9
- 10 sehr effektiv

DE03

88. Welche und wie effektiv konnten Ihre endometriosebedingten Beschwerden oder Nebenwirkungen von Medikamenten, die Sie aufgrund ihrer Endometriose einnehmen durch Dehnungsübungen in ihrer Intensität oder Dauer reduziert werden?

	starke Verbesserung (über 50%)	mäßige Verbesserung (25-50%)	geringe Verbesserung (unter 25%)	keine Verbesserung	Verschlechterung der Beschwerden	Ich habe dieses Symptom nicht
Übelkeit/ Erbrechen	○	○	○	○	○	○
Magen-/ Verdauungsbeschwerden	○	○	○	○	○	○
Müdigkeit	○	○	○	○	○	○
Angstzustände	○	○	○	○	○	○
Depression/ gedrückte Stimmung	○	○	○	○	○	○
Schlaf	○	○	○	○	○	○
Regelschmerzen/ zyklische Unterbauchschmerzen	○	○	○	○	○	○
azyklische Unterbauchschmerzen	○	○	○	○	○	○
Schmerzen beim Geschlechtsverkehr	○	○	○	○	○	○
Schmerzen beim Urin lassen	○	○	○	○	○	○
Schmerzen beim Stuhlgang	○	○	○	○	○	○

DE04

89. Haben Sie eine andere Wirkung von der Durchführung von Dehnungsübungen erwartet?

○ Ja ○ Nein

1 aktive(r) Filter

Filter DE04/F1
Wenn eine der folgenden Antwortoption(en) ausgewählt wurde: **1**
Dann Frage/Text **DE05** später im Fragebogen anzeigen (sonst ausblenden)

DE05

90. Welche Erwartungen hatten Sie bezüglich der Durchführung von Dehnungsübungen?

DE06

91. Konnte die Durchführung von Dehnungsübungen die Menge an Medikamenten, die Sie für die Therapie ihrer endometriosebedingten Symptome normalerweise benötigen reduzieren? (z.B. die Menge an Schmerzmittel)

○ Ja, starke Reduktion (über 50%) ○ Ja, mäßige Reduktion (25-50%) ○ Ja, geringe Reduktion (unter 25%) ○ Nein

92. Wie viel hat Sie durchschnittlich die Durchführung von Dehnungsübungen monatlich gekostet? DE07

(z.B. monatliche Mitgliedsgebühr, Anfängerkurse)

93. Würden Sie Dehnungsübungen einer Freundin oder einem Familienmitglied mit Endometriose weiterempfehlen? DE08

○ Ja ○ Nein

94. Gibt es etwas weiteres, was Sie uns bezüglich Dehnungsübungen mitteilen möchten? DE09

WA01

95. Wie häufig führen Sie Wärmeanwendungen durchschnittlich durch?

- ○ (mehrmals) täglich
- ○ 2-6 mal pro Woche
- ○ einmal wöchentlich
- ○ weniger als einmal wöchentlich

WA11

96. Wie ist/war die typische Schmerzstärke unter Wärmeanwendungen?

- 0 kein Schmerz
- 1
- 2
- 3
- 4
- 5
- 6
- 7
- 8
- 9
- 10 stärkster vorstellbarer Schmerz

WA10

97. Auf einer Skala von 0-10, wie effektiv würden Sie das Durchführen von Wärmeanwendungen für die Reduktion Ihrer endometriosebedingten Unterleibsschmerzen beurteilen?

- 0 überhaupt nicht effektiv
- 1
- 2
- 3
- 4
- 5
- 6
- 7
- 8
- 9
- 10 sehr effektiv

WA03

98. Welche und wie effektiv konnten Ihre endometriosebedingten Beschwerden oder Nebenwirkungen von Medikamenten, die Sie aufgrund ihrer Endometriose einnehmen, durch Wärmeanwendungen in ihrer Intensität oder Dauer reduziert werden?

	starke Verbesserung (über 50%)	mäßige Verbesserung (25-50%)	geringe Verbesserung (unter 25%)	keine Verbesserung	Verschlechterung der Beschwerden	Ich habe dieses Symptom nicht
Übelkeit/ Erbrechen	○	○	○	○	○	○
Magen-/ Verdauungsbeschwerden	○	○	○	○	○	○
Müdigkeit	○	○	○	○	○	○
Angstzustände	○	○	○	○	○	○
Depression/ gedrückte Stimmung	○	○	○	○	○	○
Schlaf	○	○	○	○	○	○
Regelschmerzen/ zyklische Unterbauchschmerzen	○	○	○	○	○	○
azyklische Unterbauchschmerzen	○	○	○	○	○	○
Schmerzen beim Geschlechtsverkehr	○	○	○	○	○	○
Schmerzen beim Urin lassen	○	○	○	○	○	○
Schmerzen beim Stuhlgang	○	○	○	○	○	○

WA04

99. Haben Sie eine andere Wirkung von der Durchführung von Wärmeanwendungen erwartet?

○ Ja ○ Nein

1 aktive(r) Filter

Filter WA04/F1
Wenn eine der folgenden Antwortoption(en) ausgewählt wurde: **1**
Dann Frage/Text **WA05** später im Fragebogen anzeigen (sonst ausblenden)

WA05

100. Welche Erwartungen hatten Sie bezüglich der Durchführung von Wärmeanwendungen?

WA06

101. Konnte die Durchführung von Wärmeanwendungen die Menge an Medikamenten, die Sie für die Therapie Ihrer endometriosebedingten Symptome normalerweise benötigen reduzieren? (z.B. die Menge an Schmerzmittel)

- ○ Ja, starke Reduktion (über 50%)
- ○ Ja, mäßige Reduktion (25-50%)
- ○ Ja, geringe Reduktion (unter 25%)
- ○ Nein

WA07

102. Wie viel hat Sie durchschnittlich die Durchführung von Wärmeanwendungen monatlich gekostet?

WA08

103. Würden Sie die Anwendung von Wärme einer Freundin oder einem Familienmitglied mit Endometriose weiterempfehlen?

- ○ Ja
- ○ Nein

WA09

104. Gibt es etwas weiteres, was Sie uns bezüglich der Anwendung von Wärme mitteilen möchten?

KA01

105. Wie häufig führen Sie Kälteanwendungen durchschnittlich durch?

○ (mehrmals) täglich ○ 2-6 mal pro Woche ○ einmal wöchentlich ○ weniger als einmal wöchentlich

KA11

106. Wie ist/war die typische Schmerzstärke unter Kälteanwendungen?

0 kein Schmerz
1
2
3
4
5
6
7
8
9
10 stärkster vorstellbarer Schmerz

KA10

107. Auf einer Skala von 0-10, wie effektiv würden Sie das Durchführen von Kälteanwendungen für die Reduktion Ihrer endometriosebedingten Unterleibsschmerzen beurteilen?

0 überhaupt nicht effektiv
1
2
3
4
5
6
7
8
9
10 sehr effektiv

KA03

108. Welche und wie effektiv konnten Ihre endometriosebedingten Beschwerden oder Nebenwirkungen von Medikamenten, die Sie aufgrund ihrer Endometriose einnehmen durch Yoga oder Pilates in ihrer Intensität oder Dauer reduziert werden?

	starke Verbesserung (über 50%)	mäßige Verbesserung (25-50%)	geringe Verbesserung (unter 25%)	keine Verbesserung	Verschlechterung der Beschwerden	Ich habe dieses Symptom nicht
Übelkeit/ Erbrechen	○	○	○	○	○	○
Magen-/ Verdauungsbeschwerden	○	○	○	○	○	○
Müdigkeit	○	○	○	○	○	○
Angstzustände	○	○	○	○	○	○
Depression/ gedrückte Stimmung	○	○	○	○	○	○
Schlaf	○	○	○	○	○	○
Regelschmerzen/ zyklische Unterbauchschmerzen	○	○	○	○	○	○
azyklische Unterbauchschmerzen	○	○	○	○	○	○
Schmerzen beim Geschlechtsverkehr	○	○	○	○	○	○
Schmerzen beim Urin lassen	○	○	○	○	○	○
Schmerzen beim Stuhlgang	○	○	○	○	○	○

KA04

109. Haben Sie eine andere Wirkung von der Durchführung von Kälteanwendungen erwartet?

○ Ja ○ Nein

1 aktive(r) Filter

Filter KA04/F1
Wenn eine der folgenden Antwortoption(en) ausgewählt wurde: **1**
Dann Frage/Text **KA05** später im Fragebogen anzeigen (sonst ausblenden)

KA05

110. Welche Erwartungen hatten Sie bezüglich der Durchführung von Kälteanwendungen?

KA06

111. Konnte die Durchführung von Kälteanwendungen die Menge an Medikamenten, die Sie für die Therapie Ihrer endometriosebedingten Symptome normalerweise benötigen reduzieren? (z.B. die Menge an Schmerzmittel)

○ Ja, starke Reduktion (über 50%)
○ Ja, mäßige Reduktion (25-50%)
○ Ja, geringe Reduktion (unter 25%)
○ Nein

112. Wie viel hat Sie durchschnittlich die Durchführung von Kälteanwendungen monatlich gekostet? KA07

113. Würden Sie die Anwendung von Kälte einer Freundin oder einem Familienmitglied mit Endometriose weiterempfehlen? KA08

○ Ja
○ Nein

114. Gibt es etwas weiteres, was Sie uns bezüglich der Anwendung von Kälte mitteilen möchten? KA09

AK01

115. Wie häufig führen Sie Akupunktur durchschnittlich durch?

- (mehrmals) täglich
- 2-6 mal pro Woche
- einmal wöchentlich
- weniger als einmal wöchentlich

AK11

116. Wie ist/war die typische Schmerzstärke unter Akupuntur?

0 kein Schmerz
1
2
3
4
5
6
7
8
9
10 stärkster vorstellbarer Schmerz

AK10

117. Auf einer Skala von 0-10, wie effektiv würden Sie das Durchführen von Akupunktur für die Reduktion [illegible] endometriosebedingten Unterleibsschmerzen beurteilen?

0 überhaupt nicht effektiv
1
2
3
4
5
6
7
8
9
10 sehr effektiv

AK03

118. Welche und wie effektiv konnten Ihre endometriosebedingten Beschwerden oder Nebenwirkungen von Medikamenten, die Sie aufgrund ihrer Endometriose einnehmen, durch Akupunktur in ihrer Intensität oder Dauer reduziert werden?

	starke Verbesserung (über 50%)	mäßige Verbesserung (25-50%)	geringe Verbesserung (unter 25%)	keine Verbesserung	Verschlechterung der Beschwerden	Ich habe dieses Symptom nicht
Übelkeit/ Erbrechen	○	○	○	○	○	○
Magen-/ Verdauungsbeschwerden	○	○	○	○	○	○
Müdigkeit	○	○	○	○	○	○
Angstzustände	○	○	○	○	○	○
Depression/ gedrückte Stimmung	○	○	○	○	○	○
Schlaf	○	○	○	○	○	○
Regelschmerzen/ zyklische Unterbauchschmerzen	○	○	○	○	○	○
azyklische Unterbauchschmerzen	○	○	○	○	○	○
Schmerzen beim Geschlechtsverkehr	○	○	○	○	○	○
Schmerzen beim Urin lassen	○	○	○	○	○	○
Schmerzen beim Stuhlgang	○	○	○	○	○	○

AK04

119. Haben Sie eine andere Wirkung von der Durchführung von Akupunktur erwartet?

○ Ja

○ Nein

1 aktive(r) Filter

Filter AK04/F1
Wenn eine der folgenden Antwortoption(en) ausgewählt wurde: **1**
Dann Frage/Text **AK05** später im Fragebogen anzeigen (sonst ausblenden)

AK05

120. Welche Erwartungen hatten Sie bezüglich der Durchführung von Akupunktur?

AK06

121. Konnte die Durchführung von Akupunktur die Menge an Medikamenten, die Sie für die Therapie Ihrer endometriosebedingten Symptome normalerweise benötigen reduzieren? (z.B. die Menge an Schmerzmittel)

○ Ja, starke Reduktion (über 50%)

○ Ja, mäßige Reduktion (25-50%)

○ Ja, geringe Reduktion (unter 25%)

○ Nein

122. Wie viel hat Sie durchschnittlich die Durchführung von Akupunktur monatlich gekostet? AK07

123. Würden Sie Akupunktur einer Freundin oder einem Familienmitglied mit Endometriose weiterempfehlen? AK08

○ Ja ○ Nein

124. Gibt es etwas weiteres, was Sie uns bezüglich Akupunktur mitteilen möchten? AK09

MA01

125. Wie häufig nutzen Sie Massagebehandlungen durchschnittlich?

○ (mehrmals) täglich
○ 2-6 mal pro Woche
○ einmal wöchentlich
○ weniger als einmal wöchentlich

MA11

126. Wie ist/war die typische Schmerzstärke unter Massage?

- 0 kein Schmerz
- 1
- 2
- 3
- 4
- 5
- 6
- 7
- 8
- 9
- 10 stärkster vorstellbarer Schmerz

MA10

127. Auf einer Skala von 0-10, wie effektiv würden Sie Massagen für die Reduktion Ihrer endometriosebedingten Unterleibsschmerzen beurteilen?

- 0 überhaupt nicht effektiv
- 1
- 2
- 3
- 4
- 5
- 6
- 7
- 8
- 9
- 10 sehr effektiv

MA03

128. Welche und wie effektiv konnten Ihre endometriosebedingten Beschwerden oder Nebenwirkungen von Medikamenten, die Sie aufgrund ihrer Endometriose einnehmen, durch Massagen in ihrer Intensität oder Dauer reduziert werden?

	starke Verbesserung (über 50%)	mäßige Verbesserung (25-50%)	geringe Verbesserung (unter 25%)	keine Verbesserung	Verschlechterung der Beschwerden	Ich habe dieses Symptom nicht
Übelkeit/ Erbrechen	○	○	○	○	○	○
Magen-/ Verdauungsbeschwerden	○	○	○	○	○	○
Müdigkeit	○	○	○	○	○	○
Angstzustände	○	○	○	○	○	○
Depression/ gedrückte Stimmung	○	○	○	○	○	○
Schlaf	○	○	○	○	○	○
Regelschmerzen/ zyklische Unterbauchschmerzen	○	○	○	○	○	○
azyklische Unterbauchschmerzen	○	○	○	○	○	○
Schmerzen beim Geschlechtsverkehr	○	○	○	○	○	○
Schmerzen beim Urin lassen	○	○	○	○	○	○
Schmerzen beim Stuhlgang	○	○	○	○	○	○

MA04

129. Haben Sie eine andere Wirkung von Massagen erwartet?

○ Ja

○ Nein

1 aktive(r) Filter

Filter MA04/F1
Wenn eine der folgenden Antwortoption(en) ausgewählt wurde: **1**
Dann Frage/Text **MA05** später im Fragebogen anzeigen (sonst ausblenden)

MA05

130. Welche Erwartungen hatten Sie bezüglich Massagen?

MA06

131. Konnten Massagen die Menge an Medikamenten, die Sie für die Therapie Ihrer endometriosebedingten Symptome normalerweise benötigen reduzieren? (z.B. die Menge an Schmerzmittel)

○ Ja, starke Reduktion (über 50%)

○ Ja, mäßige Reduktion (25-50%)

○ Ja, geringe Reduktion (unter 25%)

○ Nein

132. Wie viel haben Sie durchschnittlich die Massagebehandlungen monatlich gekostet? MA07

(z.B. monatliche Mitgliedsgebühr, Anfängerkurse)

133. Würden Sie Massagebehandlungen einer Freundin oder einem Familienmitglied mit Endometriose weiterempfehlen? MA08

○ Ja ○ Nein

134. Gibt es etwas weiteres, was Sie uns bezüglich Massagebehandlungen mitteilen möchten? MA09

OS01

135. Wie häufig führen Sie Osteopathie durchschnittlich durch?

- ○ (mehrmals) täglich
- ○ 2-6 mal pro Woche
- ○ einmal wöchentlich
- ○ weniger als einmal wöchentlich

OS10

136. Wie ist/war die typische Schmerzstärke unter Osteopathie?

0 kein Schmerz
1
2
3
4
5
6
7
8
9
10 stärkster vorstellbarer Schmerz

OS02

137. Auf einer Skala von 0-10, wie effektiv würden Sie das Durchführen von Osteopathie für die Reduktion Ihrer endometriosebedingten Unterleibsschmerzen beurteilen?

0 überhaupt nicht effektiv
1
2
3
4
5
6
7
8
9
10 sehr effektiv

OS03

138. Welche und wie effektiv konnten Ihre endometriosebedingten Beschwerden oder Nebenwirkungen von Medikamenten, die Sie aufgrund ihrer Endometriose einnehmen, durch Osteopathie in ihrer Intensität oder Dauer reduziert werden?

	starke Verbesserung (über 50%)	mäßige Verbesserung (25-50%)	geringe Verbesserung (unter 25%)	keine Verbesserung	Verschlechterung der Beschwerden	Ich habe dieses Symptom nicht
Übelkeit/ Erbrechen	○	○	○	○	○	○
Magen-/ Verdauungsbeschwerden	○	○	○	○	○	○
Müdigkeit	○	○	○	○	○	○
Angstzustände	○	○	○	○	○	○
Depression/ gedrückte Stimmung	○	○	○	○	○	○
Schlaf	○	○	○	○	○	○
Regelschmerzen/ zyklische Unterbauchschmerzen	○	○	○	○	○	○
azyklische Unterbauchschmerzen	○	○	○	○	○	○
Schmerzen beim Geschlechtsverkehr	○	○	○	○	○	○
Schmerzen beim Urin lassen	○	○	○	○	○	○
Schmerzen beim Stuhlgang	○	○	○	○	○	○

OS04

139. Haben Sie eine andere Wirkung von der Durchführung von Osteopathie erwartet?

○ Ja

○ Nein

1 aktive(r) Filter

Filter OS04/F1
[inaktiv] Keine Bedingung ausgewählt
Dann Frage/Text **OS05** später im Fragebogen anzeigen (sonst ausblenden)

OS05

140. Welche Erwartungen hatten Sie bezüglich der Durchführung von Osteopathie?

OS06

141. Konnte die Durchführung von Osteopathie die Menge an Medikamenten, die Sie für die Therapie Ihrer endometriosebedingten Symptome normalerweise benötigen reduzieren? (z.B. die Menge an Schmerzmittel)

○ Ja, starke Reduktion (über 50%)

○ Ja, mäßige Reduktion (25-50%)

○ Ja, geringe Reduktion (unter 25%)

○ Nein

OS07

142. Wie viel hat Sie durchschnittlich die Durchführung von Osteopathie monatlich gekostet? (z.B. monatliche Mitgliedsgebühr, Anfängerkurse)

OS08

143. Würden Sie Osteophatie einer Freundin oder einem Familienmitglied mit Endometriose weiterempfehlen?

○ Ja

○ Nein

OS09

144. Gibt es etwas weiteres, was Sie uns bezüglich Osteopathie mitteilen möchten?

TC01

145. Wie häufig führen Sie traditionelle chinesische Medizin durchschnittlich durch?

○ (mehrmals) täglich
○ 2-6 mal pro Woche
○ einmal wöchentlich
○ weniger als einmal wöchentlich

TC10

146. Wie ist/war die typische Schmerzstärke unter Chinesische Medizin?

- 0 kein Schmerz
- 1
- 2
- 3
- 4
- 5
- 6
- 7
- 8
- 9
- 10 stärkster vorstellbarer Schmerz

TC02

147. Auf einer Skala von 0-10, wie effektiv würden Sie das Durchführen von traditioneller chinesischer Medizin für die Reduktion Ihrer endometriosebedingten Unterleibsschmerzen beurteilen?

- 0 überhaupt nicht effektiv
- 1
- 2
- 3
- 4
- 5
- 6
- 7
- 8
- 9
- 10 sehr effektiv

TC03

148. Welche und wie effektiv konnten Ihre endometriosebedingten Beschwerden oder Nebenwirkungen von Medikamenten, die Sie aufgrund ihrer Endometriose einnehmen durch traditionelle chinesische Medizin reduziert werden?

	starke Verbesserung (über 50%)	mäßige Verbesserung (25-50%)	geringe Verbesserung (unter 25%)	keine Verbesserung	Verschlechterung der Beschwerden	Ich habe dieses Symptom nicht
Übelkeit/ Erbrechen	○	○	○	○	○	○
Magen-/ Verdauungsbeschwerden	○	○	○	○	○	○
Müdigkeit	○	○	○	○	○	○
Angstzustände	○	○	○	○	○	○
Depression/ gedrückte Stimmung	○	○	○	○	○	○
Schlaf	○	○	○	○	○	○
Regelschmerzen/ zyklische Unterbauchschmerzen	○	○	○	○	○	○
azyklische Unterbauchschmerzen	○	○	○	○	○	○
Schmerzen beim Geschlechtsverkehr	○	○	○	○	○	○
Schmerzen beim Urin lassen	○	○	○	○	○	○

TC04

149. Haben Sie eine andere Wirkung von der Durchführung von traditioneller chinesischer Medizin erwartet?

○ Ja

○ Nein

1 aktive(r) Filter

Filter TC04/F1
Wenn eine der folgenden Antwortoption(en) ausgewählt wurde: **1**
Dann Frage/Text **TC05** später im Fragebogen anzeigen (sonst ausblenden)

TC05

150. Welche Erwartungen hatten Sie bezüglich der Durchführung von traditioneller chinesischer Medizin?

TC06

151. Konnte die Durchführung von traditioneller chinesischer Medizin die Menge an Medikamenten, die Sie für die Therapie Ihrer endometriosebedingten Symptome normalerweise benötigen reduzieren? (z.B. die Menge an Schmerzmittel)

○ Ja, starke Reduktion (über 50%)

○ Ja, mäßige Reduktion (25-50%)

○ Ja, geringe Reduktion (unter 25%)

○ Nein

TC07

152. Wie viel hat Sie durchschnittlich die Durchführung von traditioneller chinesischer Medizin monatlich gekostet?

(z.B. monatliche Mitgliedsgebühr, Anfängerkurse)

TC08

153. Würden Sie traditionelle chinesische Medizin einer Freundin oder einem Familienmitglied mit Endometriose weiterempfehlen?

Ja

Nein

TC09

154. Gibt es etwas weiteres, was Sie uns bezüglich traditioneller chinesischer Medizin mitteilen möchten?

AL01

155. Wie häufig konsumieren Sie Alkohol durchschnittlich?

○ (mehrmals) täglich
○ 2-6 mal pro Woche
○ einmal wöchentlich
○ weniger als einmal wöchentlich

AL11

156. Wie ist/war die typische Schmerzstärke unter Alkohol?

0 kein Schmerz
1
2
3
4
5
6
7
8
9
10 stärkster vorstellbarer Schmerz

AL10

157. Auf einer Skala von 0-10, wie effektiv würden Sie den Konsum von Alkohol für die Reduktion Ihrer endometriosebedingten Unterleibsschmerzen beurteilen?

0 überhaupt nicht effektiv
1
2
3
4
5
6
7
8
9
10 sehr effektiv

AL03

158. Welche und wie effektiv konnten Ihre endometriosebedingten Beschwerden oder Nebenwirkungen von Medikamenten, die Sie aufgrund ihrer Endometriose einnehmen, durch den Konsum von Alkohol in ihrer Intensität oder Dauer reduziert werden?

	starke Verbesserung (über 50%)	mäßige Verbesserung (25-50%)	geringe Verbesserung (unter 25%)	keine Verbesserung	Verschlechterung der Beschwerden	Ich habe dieses Symptom nicht
Übelkeit/ Erbrechen	◯	◯	◯	◯	◯	◯
Magen-/ Verdauungsbeschwerden	◯	◯	◯	◯	◯	◯
Müdigkeit	◯	◯	◯	◯	◯	◯
Angstzustände	◯	◯	◯	◯	◯	◯
Depression/ gedrückte Stimmung	◯	◯	◯	◯	◯	◯
Schlaf	◯	◯	◯	◯	◯	◯
Regelschmerzen/ zyklische Unterbauchschmerzen	◯	◯	◯	◯	◯	◯
azyklische Unterbauchschmerzen	◯	◯	◯	◯	◯	◯
Schmerzen beim Geschlechtsverkehr	◯	◯	◯	◯	◯	◯
Schmerzen beim Urin lassen	◯	◯	◯	◯	◯	◯
Schmerzen beim Stuhlgang	◯	◯	◯	◯	◯	◯

AL04

159. Haben Sie eine andere Wirkung von dem Konsum von Alkohol erwartet?

◯ Ja ◯ Nein

1 aktive(r) Filter

Filter AL04/F1
Wenn eine der folgenden Antwortoption(en) ausgewählt wurde: **1**
Dann Frage/Text **AL05** später im Fragebogen anzeigen (sonst ausblenden)

AL05

160. Welche Erwartungen hatten Sie bezüglich dem Konsum von Alkohol?

AL06

161. Konnte der Konsum von Alkohol die Menge an Medikamenten, die Sie für die Therapie Ihrer endometriosebedingten Symptome normalerweise benötigen reduzieren? (z.B. die Menge an Schmerzmittel)

◯ Ja, starke Reduktion (über 50%) ◯ Ja, mäßige Reduktion (25-50%) ◯ Ja, geringe Reduktion (unter 25%) ◯ Nein

162. Wie viel hat Sie durchschnittlich der Konsum von Alkohol monatlich gekostet? AL07

(z.B. monatliche Mitgliedsgebühr, Anfängerkurse)

163. Würden Sie den Konsum von Alkohol einer Freundin oder einem Familienmitglied mit Endometriose weiterempfehlen? AL08

Ja

Nein

164. Gibt es etwas weiteres, was Sie uns bezüglich des Konsums von Alkohol mitteilen möchten? AL09

NA01

165. Wie häufig nehmen Sie natürliche Beruhigungsmittel durchschnittlich ein?

○	○	○	○
(mehrmals) täglich	2-6 mal pro Woche	einmal wöchentlich	weniger als einmal wöchentlich

NA11

166. Wie ist/war die typische Schmerzstärke unter natürlichen Beruhigungsmittel?

- 0 kein Schmerz
- 1
- 2
- 3
- 4
- 5
- 6
- 7
- 8
- 9
- 10 stärkster vorstellbarer Schmerz

NA10

167. Auf einer Skala von 0-10, wie effektiv würden Sie das Einnehmen von natürlichen Beruhigungsmitteln für die Reduktion Ihrer endometriosebedingten Unterleibsschmerzen beurteilen?

- 0 überhaupt nicht effektiv
- 1
- 2
- 3
- 4
- 5
- 6
- 7
- 8
- 9
- 10 sehr effektiv

NA03

168. Welche und wie effektiv konnten Ihre endometriosebedingten Beschwerden oder Nebenwirkungen von Medikamenten, die Sie aufgrund ihrer Endometriose einnehmen, durch natürliche Beruhigungsmittel in ihrer Intensität oder Dauer reduziert werden?

	starke Verbesserung (über 50%)	mäßige Verbesserung (25-50%)	geringe Verbesserung (unter 25%)	keine Verbesserung	Verschlechterung der Beschwerden	Ich habe dieses Symptom nicht
Übelkeit/ Erbrechen	○	○	○	○	○	○
Magen-/ Verdauungsbeschwerden	○	○	○	○	○	○
Müdigkeit	○	○	○	○	○	○
Angstzustände	○	○	○	○	○	○
Depression/ gedrückte Stimmung	○	○	○	○	○	○
Schlaf	○	○	○	○	○	○
Regelschmerzen/ zyklische Unterbauchschmerzen	○	○	○	○	○	○
azyklische Unterbauchschmerzen	○	○	○	○	○	○
Schmerzen beim Geschlechtsverkehr	○	○	○	○	○	○
Schmerzen beim Urin lassen	○	○	○	○	○	○
Schmerzen beim Stuhlgang	○	○	○	○	○	○

NA04

169. Haben Sie eine andere Wirkung von der Einnahme von natürlichen Beruhigungsmitteln erwartet?

○ Ja

○ Nein

1 aktive(r) Filter

Filter NA04/F1
Wenn eine der folgenden Antwortoption(en) ausgewählt wurde: **1**
Dann Frage/Text **NA05** später im Fragebogen anzeigen (sonst ausblenden)

NA05

170. Welche Erwartungen hatten Sie bezüglich der Einnahme von natürliche Beruhigungsmitteln?

NA06

171. Konnte die Einnahme von natürlichen Beruhigungsmitteln die Menge an Medikamenten, die Sie für die Therapie Ihrer endometriosebedingten Symptome normalerweise benötigen reduzieren? (z.B. die Menge an Schmerzmittel)

- ○ Ja, starke Reduktion (über 50%)
- ○ Ja, mäßige Reduktion (25-50%)
- ○ Ja, geringe Reduktion (unter 25%)
- ○ Nein

NA07

172. Wie viel hat Sie durchschnittlich die Einnahme von natürlichen Beruhigungsmitteln monatlich gekostet?

NA08

173. Würden Sie natürliche Beruhigungsmittel einer Freundin oder einem Familienmitglied mit Endometriose weiterempfehlen?

- ○ Ja
- ○ Nein

NA09

174. Gibt es etwas weiteres, was Sie uns bezüglich der Einnahme von natürlichen Beruhigungsmitteln mitteilen möchten?

ER01

175. Wie häufig ernähren Sie dich durchschnittlich nach einer bestimmten Diät oder Ernährungsform (z.B. Paleo-Diät, pflanzenbasierte Kost, FODMAP-Diät)?

- ○ (mehrmals) täglich
- ○ 2-6 mal pro Woche
- ○ einmal wöchentlich
- ○ weniger als einmal wöchentlich

ER11

176. Wie ist/war die typische Schmerzstärke unter Ernährung?

- 0 kein Schmerz
- 1
- 2
- 3
- 4
- 5
- 6
- 7
- 8
- 9
- 10 stärkster vorstellbarer Schmerz

ER10

177. Auf einer Skala von 0-10, wie effektiv würden Sie Ihre Ernährungsweise für die Reduktion Ihrer endometriosebedingten Unterleibsschmerzen beurteilen?

- 0 überhaupt nicht effektiv
- 1
- 2
- 3
- 4
- 5
- 6
- 7
- 8
- 9
- 10 sehr effektiv

ER03

178. Welche und wie effektiv konnten Ihre endometriosebedingten Beschwerden oder Nebenwirkungen von Medikamenten, die Sie aufgrund ihrer Endometriose einnehmen, durch Ihre Ernährungsweise in ihrer Intensität oder Dauer reduziert werden?

	starke Verbesserung (über 50%)	mäßige Verbesserung (25-50%)	geringe Verbesserung (unter 25%)	keine Verbesserung	Verschlechterung der Beschwerden	Ich habe dieses Symptom nicht
Übelkeit/ Erbrechen	○	○	○	○	○	○
Magen-/ Verdauungsbeschwerden	○	○	○	○	○	○
Müdigkeit	○	○	○	○	○	○
Angstzustände	○	○	○	○	○	○
Depression/ gedrückte Stimmung	○	○	○	○	○	○
Schlaf	○	○	○	○	○	○
Regelschmerzen/ zyklische Unterbauchschmerzen	○	○	○	○	○	○
azyklische Unterbauchschmerzen	○	○	○	○	○	○
Schmerzen beim Geschlechtsverkehr	○	○	○	○	○	○
Schmerzen beim Urin lassen	○	○	○	○	○	○
Schmerzen beim Stuhlgang	○	○	○	○	○	○

ER04

179. Haben Sie eine andere Wirkung von Ihrer Ernährungsweise erwartet?

○ Ja

○ Nein

1 aktive(r) Filter

Filter ER04/F1
Wenn eine der folgenden Antwortoption(en) ausgewählt wurde: **1**
Dann Frage/Text **ER05** später im Fragebogen anzeigen (sonst ausblenden)

ER05

180. Welche Erwartungen hatten Sie bezüglich Ihrer Ernährungsweise?

ER06

181. Konnte Ihre Ernährungsweise die Menge an Medikamenten, die Sie für die Therapie Ihrer endometriosebedingten Symptome normalerweise benötigen reduzieren? (z.B. die Menge an Schmerzmittel)

○ Ja, starke Reduktion (über 50%)

○ Ja, mäßige Reduktion (25-50%)

○ Ja, geringe Reduktion (unter 25%)

○ Nein

182. Wie viel hat Sie durchschnittlich Ihre Ernährungsweise monatlich gekostet? ER07

bitte die Kosten angeben, die über Ihre normalen Lebensmittelkosten hinaus entstanden sind

183. Würden Sie Ihre Ernährungsweise einer Freundin oder einem Familienmitglied mit Endometriose weiterempfehlen? ER08

○ Ja ○ Nein

184. Gibt es etwas weiteres, was Sie uns bezüglich Ihrer Ernährungsweise mitteilen möchten? ER09

OL01

185. Wie häufig nehmen Sie Hanf-/CBD-Öl durchschnittlich ein?

○ (mehrmals) täglich
○ 2-6 mal pro Woche
○ einmal wöchentlich
○ weniger als einmal wöchentlich

OL11

186. Wie ist/war die typische Schmerzstärke unter Hanf-/CBD-Öl?

- 0 kein Schmerz
- 1
- 2
- 3
- 4
- 5
- 6
- 7
- 8
- 9
- 10 stärkster vorstellbarer Schmerz

OL10

187. Auf einer Skala von 0-10, wie effektiv würden Sie die Einnahme von Hanf-/CBD-Öl für die Reduktion Ihrer endometriosebedingten Unterleibsschmerzen beurteilen?

- 0 überhaupt nicht effektiv
- 1
- 2
- 3
- 4
- 5
- 6
- 7
- 8
- 9
- 10 sehr effektiv

OL03

188. Welche und wie effektiv konnten Ihre endometriosebedingten Beschwerden oder Nebenwirkungen von Medikamenten, die Sie aufgrund ihrer Endometriose einnehmen, durch die Einnahme von Hanf-/CBD-Öl in ihrer Intensität oder Dauer reduziert werden?

	starke Verbesserung (über 50%)	mäßige Verbesserung (25-50%)	geringe Verbesserung (unter 25%)	keine Verbesserung	Verschlechterung der Beschwerden	Ich habe dieses Symptom nicht
Übelkeit/ Erbrechen	○	○	○	○	○	○
Magen-/ Verdauungsbeschwerden	○	○	○	○	○	○
Müdigkeit	○	○	○	○	○	○
Angstzustände	○	○	○	○	○	○
Depression/ gedrückte Stimmung	○	○	○	○	○	○
Schlaf	○	○	○	○	○	○
Regelschmerzen/ zyklische Unterbauchschmerzen	○	○	○	○	○	○
azyklische Unterbauchschmerzen	○	○	○	○	○	○
Schmerzen beim Geschlechtsverkehr	○	○	○	○	○	○
Schmerzen beim Urin lassen	○	○	○	○	○	○
Schmerzen beim Stuhlgang	○	○	○	○	○	○

189. Haben Sie eine andere Wirkung von der Einnahme von Hanf-/CBD-Öl erwartet? OL04

○ Ja ○ Nein

1 aktive(r) Filter

Filter OL04/F1
Wenn eine der folgenden Antwortoption(en) ausgewählt wurde: **1**
Dann Frage/Text **OL05** später im Fragebogen anzeigen (sonst ausblenden)

190. Welche Erwartungen hatten Sie bezüglich der Einnahme von Hanf-/CBD-Öl? OL05

191. Konnte die Einnahme von Hanf-/CBD-Öl die Menge an Medikamenten, die Sie für die Therapie Ihrer endometriosebedingten Symptome normalerweise benötigen reduzieren? (z.B. die Menge an Schmerzmittel) OL06

○ Ja, starke Reduktion (über 50%)
○ Ja, mäßige Reduktion (25-50%)
○ Ja, geringe Reduktion (unter 25%)
○ Nein

192. Wie viel hat Sie durchschnittlich die Einnahme von Hanf-/CBD-Öl monatlich gekostet? OL07

193. Würden Sie Hanf-/CBD-Öl einer Freundin oder einem Familienmitglied mit Endometriose weiterempfehlen? OL08

○ Ja

○ Nein

194. Gibt es etwas weiteres, was Sie uns bezüglich Einnahme von Hanf-/CBD-Öl mitteilen möchten? OL09

CA01

195. Wie häufig konsumieren Sie Cannabis in den folgenden Formen durchschnittlich?

	(mehrmals) täglich	2-6 mal pro Woche	einmal wöchentlich	weniger als einmal wöchentlich
Rauchen	○	○	○	○
Sisha/ Wasserpfeife	○	○	○	○
in Lebensmitteln (Kekse, Brownies,...)	○	○	○	○
Sonstige	○	○	○	○

CA11

196. Wie ist/war die typische Schmerzstärke unter Cannabis?

0 kein Schmerz
1
2
3
4
5
6
7
8
9
10 stärkster vorstellbarer Schmerz

CA10

197. Auf einer Skala von 0-10, wie effektiv würden Sie den Konsum von Cannabis für die Reduktion Ihrer endometriosebedingten Unterleibsschmerzen beurteilen?

0 überhaupt nicht effektiv
1
2
3
4
5
6
7
8
9
10 sehr effektiv

CA03

198. Welche und wie effektiv konnten Ihre endometriosebedingten Beschwerden oder Nebenwirkungen von Medikamenten, die Sie aufgrund ihrer Endometriose einnehmen, durch den Konsum von Cannabis in ihrer Intensität oder Dauer reduziert werden?

	starke Verbesserung (über 50%)	mäßige Verbesserung (25-50%)	geringe Verbesserung (unter 25%)	keine Verbesserung	Verschlechterung der Beschwerden	Ich habe dieses Symptom nicht
Übelkeit/ Erbrechen	○	○	○	○	○	○
Magen-/ Verdauungsbeschwerden	○	○	○	○	○	○
Müdigkeit	○	○	○	○	○	○
Angstzustände	○	○	○	○	○	○
Depression/ gedrückte Stimmung	○	○	○	○	○	○
Schlaf	○	○	○	○	○	○
Regelschmerzen/ zyklische Unterbauchschmerzen	○	○	○	○	○	○
azyklische Unterbauchschmerzen	○	○	○	○	○	○
Schmerzen beim Geschlechtsverkehr	○	○	○	○	○	○
Schmerzen beim Urin lassen	○	○	○	○	○	○
Schmerzen beim Stuhlgang	○	○	○	○	○	○

CA04

199. Haben Sie eine andere Wirkung von dem Konsum von Cannabis erwartet?

○ Ja ○ Nein

1 aktive(r) Filter

Filter CA04/F1
Wenn eine der folgenden Antwortoption(en) ausgewählt wurde: **1**
Dann Frage/Text **CA05** später im Fragebogen anzeigen (sonst ausblenden)

CA05

200. Welche Erwartungen hatten Sie bezüglich des Konsums von Cannabis?

CA06

201. Konnte den Konsum von Cannabis die Menge an Medikamenten, die Sie für die Therapie Ihrer endometriosebedingten Symptome normalerweise benötigen reduzieren? (z.B. die Menge an Schmerzmittel)

○ Ja, starke Reduktion (über 50%) ○ Ja, mäßige Reduktion (25-50%) ○ Ja, geringe Reduktion (unter 25%) ○ Nein

202. Wie viel hat Sie durchschnittlich der Konsum von Cannabis monatlich gekostet? CA07

203. Würden Sie Cannabis einer Freundin oder einem Familienmitglied mit Endometriose weiterempfehlen? CA08

○ Ja ○ Nein

204. Gibt es etwas weiteres, was Sie uns bezüglich des Konsums von Cannabis mitteilen möchten? CA09

KL01

205. Würden Sie die folgenden Substanzen für die Bewältigung Ihrer endometriosebedingten Schmerzen ausprobieren wollen?

	Ja	Nein
Cannabidiol – CBD	○	○
Tetrahydrocannabinol – THC	○	○

KL02

206. Würden Sie die folgenden Substanzen für die Bewältigung Ihrer endometriosebedingten Schmerzen ausprobieren wollen, wenn diese von Ihrem Arzt rezeptpflichtig verschrieben werden würden?

	Ja	Nein
Cannabidiol – CBD	○	○
Tetrahydrocannabinol – THC	○	○

KL03

207. In welcher Verabreichungsform würden Sie CBD einnehmen?

	Ja	Nein
oral (z.B als Tablette, Tropfen)	○	○
sublingual/unter der Zunge	○	○
Hautpflaster	○	○
Creme/Gel	○	○
Scheidenzäpfchen	○	○
weitere	○	○

KL04

208. In welcher Verabreichungsform würden Sie THC einnehmen?

	Ja	Nein
oral (z.B als Tablette, Tropfen)	○	○
sublingual/unter der Zunge	○	○
Hautpflaster	○	○
Creme/Gel	○	○
Scheidenzäpfchen	○	○
weitere	○	○

KL05

209. Wären Sie daran interessiert an einer klinischen Studie teilzunehmen, die die Einnahme folgender Substanzen beinhaltet und untersucht?

	Ja	Nein
CBD	○	○
THC	○	○
CBD + THC in Kombination	○	○

Letzte Seite

Vielen Dank für Ihre Teilnahme!

Wir möchten uns ganz herzlich für Ihre Mithilfe bedanken.

Ihre Antworten wurden gespeichert, Sie können das Browser-Fenster nun schließen.

Dr. Sylvia Mechsner und Renata Voltolini Velho, PhD Endometriosezentrum, Charité - Universitätsmedizin Berlin – 2022
